# Key personality and training factors influencing athletes’ mental health - based on machine learning

**DOI:** 10.1371/journal.pone.0335918

**Published:** 2025-12-17

**Authors:** Guoxiao Sun, Jun Wang, Shuangling Zhang

**Affiliations:** 1 School of Physical Education, Shandong University, Jinan, China; 2 Shandong Institute of Sport Science, Jinan, China; King Khalid University, EGYPT

## Abstract

Athletes face a higher risk of mental health disorders compared to the general population, and prior theoretical and empirical work suggests that personality traits and training-related factors may play important roles in shaping athletes’ mental health. This study aimed to identify the key personality traits and training-related factors associated with mental health outcomes among athletes. A total of 328 athletes (53% male, 17.84 ± 3.155 years) were assessed using standardized measures of demographics, training history, Cattell’s 16 Personality Factors, and mental health status, with psychological symptoms evaluated via the Symptom Checklist-90 (SCL-90). To determine the most influential factors related to mental disorders, both random forest and logistic regression models were applied. The results showed that 44.6% of athletes presented at least one symptom of a mental disorder. According to the random forest model, the most significant personality and training factors included injury impact, athletic level, training years, boldness, sensitivity, apprehension, and tension. Logistic regression analysis further indicated that high boldness, higher athletic level, and longer training years acted as protective factors, while greater injury impact, apprehension, tension, and sensitivity were associated with an increased risk of mental disorders. These results identify key risk and protective factors for mental health disorders among athletes, highlighting the need for individualized approaches in athlete mental health management, and emphasizing the importance of early identification and targeted interventions.

## 1. Introduction

The mental health of athletes has continued to deteriorate in recent years, with elite athletes dropping out of competitions, performing poorly, and even committing suicide due to mental disorders [1]. In 2018, the International Olympic Committee (IOC) held the ‘Consensus Meeting on Mental Health in Elite Athletes’, which placed significant emphasis on the well-being and mental health of athletes. This conference resulted in the formulation of several policies and guidelines designed to support athletes’ mental health [2]. Additionally, the International Society of Sports Psychology (ISSP), coaches, administrators, and researchers are also placing increased emphasis and attention on the mental health of athletes [3,4]. In highly stressful and competitive training and competition, athletes are subjected to psychological and physical stress and high-intensity stimuli that are difficult to experience by the general public. Furthermore, they often face difficulties in adapting to and losing interest in their sport, as well as both emotional and physical exhaustion [5]. Mental health is a crucial component of the elite athlete system and is a key indicator of effective athlete management [6]. Therefore, it is of paramount importance to improve the mental health of athletes who are subjected to significant physical and psychological stress and to eliminate the potential for mental disorders in current athlete management.

Despite the protective and ameliorative effects of exercise on mental health, research indicates that athletes are at an increased risk of developing mild to severe mental disorders compared to the general population [7]. Early screening for mental health disorders is essential, as 58% of athletes’ mental disorders remain undetected and untreated due to challenges in accessing appropriate medical diagnosis [8]. Mental disorders directly affect an athlete’s performance, resulting in diminished ability, increased injury risk, and even premature retirement [9,10]. They also critically impact their quality of life, leading to loss of interest, fatigue, reduced energy, and altered sleep patterns, which may escalate to worse outcomes such as anxiety, depression, eating disorders, and even self-harm or suicidal behavior [11,12]. Mental disorders arise from a complex interplay of social, psychological, and biological factors, including sociodemographic, family, health, and economic factors [13,14]. Recent research, grounded in personality theory and stress-vulnerability models, has increasingly highlighted the critical roles of personality traits and training-related factors in shaping athletes’ mental health. For instance, neuroticism—a core dimension in the Five-Factor Model of personality—is significantly and positively associated with anxiety and stress [15], while multiple factors identified by Cattell’s 16 Personality Factors (16PF) are linked to depressive symptoms among athletes [16]. According to these theoretical frameworks, individual differences in personality may influence vulnerability to psychological distress when faced with stressors common in competitive sports. In addition to personality, training and competition-related factors—such as sport type, competitive level, injury experience, career transitions, and major life changes—are also recognized as important determinants of athletes’ mental health outcomes [17–19]. Overall, the literature indicates that personality traits and training-related factors are the two principal domains shaping mental health outcomes in athletes. Comprehensive assessment of both the relative importance and the specific impact of these factors is critical for the development of effective, targeted interventions to enhance athlete well-being.

Prior studies often suffer from methodological limitations, as conventional statistical approaches frequently examine personality traits and training-related factors in isolation or with limited control for covariates, thereby overlooking the complex, multifactorial nature of athletes’ psychological outcomes and hindering the determination of the relative importance and specific impact of these factors on mental health.With the advent of high-dimensional data in psychological and sociological research, there is a growing need for advanced analytic approaches capable of systematically evaluating and ranking multiple predictors of mental health outcomes [20]. Variable importance analysis enables researchers to identify, validate, and prioritize key risk factors, thereby strengthening both theoretical understanding and policy formulation [21]. Machine learning methods—such as random forests, neural networks, and support vector machines—provide powerful tools for handling complex, high-dimensional datasets and uncovering nonlinear relationships among predictors [22,23]. In this multifactorial and high-dimensional context, machine learning offers distinct advantages for accurately identifying and ranking key predictors, facilitating more precise and nuanced risk assessments.

In summary, a critical gap remains in the literature regarding the integrated evaluation of personality traits and training-related factors on athletes’ mental health within a robust, data-driven framework. To address this gap, the present study combines machine learning techniques with logistic regression to systematically assess the relative contributions of these factors. The findings aim to offer clearer theoretical guidance for mental health policy prioritization and support the development of precise, individualized interventions to enhance athletes’ well-being and performance.

## 2. Method

### 2.1. Participants

This study, initiated by the Shandong Provincial Sports Bureau in June 2024, retrospectively analyzed data from 346 athletes who participated in official provincial training programs between June 2022 and May 2023. These programs, open to athletes from various sports disciplines, age groups, and training centers across Shandong Province, represent the primary pathway for competitive athletes in the region. Although direct comparison with the national athlete registry was not possible due to data access limitations, key demographic characteristics (age and gender distribution) were examined against findings reported in relevant published literature [24,25]. No significant differences were observed in gender distribution (χ² = 0.32, p = 0.57), while the age distribution was slightly younger than previous studies (mean difference = −0.42 years, p = 0.21), consistent with current recruitment practices favoring younger athletes. Overall, the sample is considered representative of athletes in Shandong’s official training programs.

During data cleaning, cases with substantial missing data—specifically those lacking key variables such as psychological health measures, personality assessments, or core training-related information—were excluded, resulting in the removal of 18 participants. For the remaining dataset, minor missing values in non-essential variables (e.g., secondary training details or supplementary questionnaire items) were addressed using mean imputation for continuous variables and mode imputation for categorical variables, ensuring data completeness and minimizing the impact of missing data on study results. As a result, a final sample of 328 athletes was included in the main analysis; of these, 53% were male, with a mean age of 17.84 ± 3.16 years.

All participants provided written informed consent prior to data collection, with procedures including a detailed explanation of study aims, data usage, potential risks, and privacy protection measures. Participants were informed of their right to withdraw at any time without consequence. The study protocol was approved by the Ethics Committee of the Preventive Medicine Research Program of Shandong University (20190609) and conducted in accordance with the Declaration of Helsinki. All data were anonymized prior to analysis, and the authors did not have access to any identifying information at any stage, ensuring participant privacy and confidentiality.

### 2.2. Variable design

#### 2.2.1. Dependent variables.

The self-rating scale (SCL-90) developed by Derogatis et al. was selected as an indicator of athletes’ mental health [26], consisting of nine factors: somatization, obsessive-compulsive, interpersonal sensitivity, depression, anxiety, hostility, phobic anxiety, paranoid ideation, psychoticism. Each item is rated on a scale ranging from 1 (not at all) to 5 (very severe), and subjects are asked to rate their feelings based on the past week, with higher mean scores on the items of each dimension being associated with a higher degree of that symptom, defining a mean score of ≥2 on the item as the presence of that symptom [27]. The SCL-90 has been widely used and validated in various populations in China, including among young athletes, demonstrating acceptable reliability and validity [24,25].

#### 2.2.2. Independent variables.

Based on previous research on factors influencing mental disorders in athletes, thirty variables were selected and organized into three domains: demographic factors, training-related factors, and personality traits assessed by Cattell’s 16 Personality Questionnaire. Each domain reflects distinct mechanisms underlying the development of mental disorders in athletes [13–19]. Demographic factors included age, gender, whether they were an only child, family form, and socioeconomic status (SES). Training-related factors included sport type, professional training experience, years of training, athlete level, whether they changed sports, whether they chose their sport, degree of impact of sports injuries, degree of recovery from injuries, and confidence in athletic achievement gained. Cattell personality factors were measured by Cattell’s 16 Personality Questionnaire and included warmth, reasoning, emotional stability, dominance, liveliness, rule-consciousness, boldness, sensitivity, vigilance, abstractedness, privateness, apprehension, openness to change, self-reliance, perfectionism and tension [28], specific classifications and codes are detailed in Table 1.

**Table 1:** Variable assignment and descriptive statistics.

Factors type	Factors	Assignment	M ± SD
Mental health	Depression	No = 0, Yes = 1	0.225
	Anxiety		0.229
	Somatization		0.133
	Obsessive-compulsive		0.375
	Interpersonal sensitivity		0.263
Demographic	Age		17.84 ± 3.155
	Gender	Female = 0, Male = 1	0.534
	SES	Poor = 1, Fair = 2, Good = 3, Very Good = 4	2.342 ± 0.712
	Only child	No = 0, Yes = 1	0.255
	Family form	Other(Single-parent or blended family)=0, Two-parent family = 1	0.825
Training-related	Sports type	Individual = 1, Group = 2	1.31
	Professional training	No = 0, Yes = 1	0.912
	Training years	0-5 = 1, 6-10 = 2, > 11 = 3	1.822 ± 0.731
	Athlete level	Other = 1, 2nd Level = 2, 1st Level = 3, Master = 4, International Master = 5	2.553 ± 1.163
	Sports change	No = 0, Yes = 1	0.119
	Self-selection	No = 0, Yes = 1	0.876
	Injury Impact	Very bad = 1, Bad = 2, Fair = 3, Good = 4, Very good = 5	3.263 ± 1.003
	Injury recovery	Very bad = 1, Bad = 2, Fair = 3, Good = 4, Very good = 5	3.612 ± 0.822
	Confidence achievement	Very bad = 1, Bad = 2, Fair = 3, Good = 4, Very good = 5	3.578 ± 0.926
Cattell Personality	Warmth		6.033 ± 1.569
	Reasoning		4.773 ± 1.667
	Emotional Stability		4.887 ± 1.656
	Dominance		4.442 ± 1.526
	Liveliness		5.677 ± 1.637
	Rule-Consciousness		4.672 ± 1.303
	Boldness		5.558 ± 1.731
	Sensitivity		6.342 ± 1.432
	Vigilance		4.226 ± 1.503
	Abstractedness		4.891 ± 1.354
	Privateness		5.585 ± 1.362
	Apprehension		5.757 ± 1.645
	Openness to Change		4.653 ± 1.335
	Self-Reliance		5.063 ± 1.516
	Perfectionism		5.087 ± 1.292
	Tension		6.201 ± 1.332

### 2.3. Assessment of the importance of factors

#### 2.3.1. Random forest model importance assessment.

The Random Forest (RF) algorithm, introduced by Breiman [29], marks a significant advancement in ensemble machine learning, especially in fields such as psychology and sociology, where data complexity and high dimensionality often pose challenges to traditional modeling approaches. Unlike linear models, which depend on strict assumptions about data distributions and model structures, Random Forests are non-parametric and highly flexible. This flexibility enables RF to effectively capture nonlinear relationships, variable interactions, and to accommodate both high-dimensional and categorical data [30]. Moreover, Random Forests exhibit strong robustness to noise and overfitting, making them a reliable tool for empirical research in the social sciences [31].

A central advantage of Random Forests is their capacity to assess the importance of variables within an ensemble framework. This is most commonly achieved through the Mean Decrease Impurity (MDI) method, which quantifies each predictor’s contribution by measuring the reduction in node impurity—such as Gini impurity or entropy—across all decision trees in the forest. When a variable is used to split a node, it partitions the data to increase homogeneity in the resulting subsets, thereby reducing impurity. By averaging these reductions across all trees, the MDI method yields a relative ranking of variable importance. Variables associated with greater average impurity reductions are considered more influential in accurate classification or prediction [32]. In this study, the MDI method was employed to systematically evaluate the relative importance of factors affecting depressive symptoms.

#### 2.3.2. Importance assessment stability test and selection of key factors.

The Random Forest MDI algorithm is a commonly used method for assessing the importance of variables. It has many advantages in assessing the relative importance of factors, but also disadvantages that should not be ignored. These include the possibility of overestimating the importance of factors due to a large range of values [33]. To verify the validity and stability of the MDI method for ranking the importance of factors influencing mental health, the study introduced a stability test utilizing the Mean Decrease Accuracy (MDA) method.

The MDA method assesses feature importance by calculating the influence of a variable on the prediction accuracy of the dependent variable in a random forest model, randomly disrupting the value of a particular variable and calculating the difference in model accuracy after the disruption; the greater the decrease in model prediction accuracy, the higher the importance of the variable. Checking whether the MDA and MDI algorithms are overall the same in ranking the importance of the variables, if the rankings are roughly the same then it shows that the ranking of variable importance using the MDI method in this study is effective and stable [34]. The MDI method emphasizes a variable’s internal importance within the model, while the MDA method focuses on its impact on predictive accuracy. This complementary approach provides a comprehensive perspective for understanding and evaluating the multifaceted factors influencing athletes’ mental health.

In this study, we considered the factors that had higher importance among the selected variables and contributed more to the prediction accuracy of depressive symptoms as important influences on athletes’ mental health. To ensure that the factors we focused on had a greater impact on the results and were of higher importance, we set the following criteria: (i) the top 10 most important factors in terms of the MDI importance rankings; and (ii) the MDA was greater than 0.001.

#### 2.3.3. Parameter setting and model stability.

**Fig 1:**
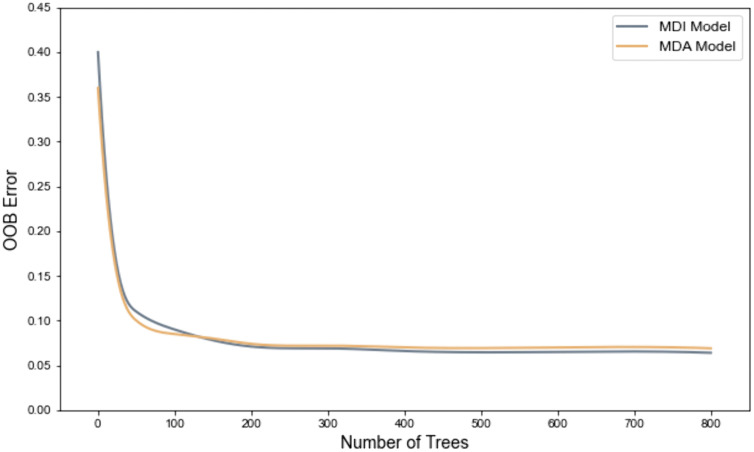
OOB error curves for MDI and MDA random forest models.

To assess the robustness and generalization capability of the Random Forest models, we first examined the out-of-bag (OOB) error curves for both the MDI and MDA models. The OOB error, an internal cross-validation metric unique to Random Forests, provides an unbiased estimate of prediction error without the need for a separate validation set. As shown in Fig 1, the OOB error for both models decreases rapidly with increasing number of trees and stabilizes once the number exceeds approximately 400. This trend aligns with prior research [35], indicating that the models have effectively learned the underlying data patterns, and further increasing the number of trees yields only marginal improvements in error reduction. The close alignment of the OOB error curves for the MDI and MDA models further demonstrates the consistency and reliability of the variable importance rankings. The convergence of the final OOB errors to a low and stable value suggests that the models are well-calibrated and possess strong generalization performance.

Based on these observations, model parameters were configured to balance performance and computational efficiency. Specifically, the number of trees (n_estimators) was set to 400, as stability is generally achieved beyond this threshold. The minimum number of samples per leaf (min_samples_leaf) was set to 5 to ensure an optimal trade-off between model fit and generalization. Maximum tree depth (max_depth) was left unrestricted to allow the model to capture complex, non-linear relationships within the data. In addition, parallel computing was employed to improve training efficiency. The random forest models were developed in Python 3.9 with the scikit-learn machine learning library, leveraging its robust implementation for parameter tuning and parallel computation.

### 2.4. Logistic regression analysis

In this study, we first utilized a random forest model to identify key personality and training factors by ranking the importance of variables for each dimension of mental disorder in athletes. These important predictors, as determined by the random forest algorithm, were subsequently incorporated into binary logistic regression models for each dimension of mental disorder, with the dependent variable defined as the presence or absence of the specific mental disorder dimension according to established criteria. Demographic factors, including age, gender, socioeconomic status (SES), only child status, and family form, were included as covariates in all logistic regression models, and all predictors were entered simultaneously. Multicollinearity among predictors was assessed using variance inflation factors (VIFs), and variables with high collinearity were excluded where necessary. All descriptive statistics, logistic regression analyses, and Harman’s single-factor test for common method bias were performed using SPSS version 26.0.

## 3. Results

### 3.1. Common method bias test

Given that all data in this study were collected through athletes’ self-report questionnaires, procedural remedies such as anonymous participation and strict confidentiality measures were implemented to mitigate the risk of common method bias. To statistically assess this potential bias, Harman’s single-factor test was performed on all survey items. The results revealed that six factors had eigenvalues greater than 1, and the first unrotated factor accounted for 28.65% of the total variance, which is well below the commonly accepted threshold of 40%. These findings indicate that common method bias is not a significant concern in this study, and the self-report nature of the data does not substantially threaten the validity of the results.

### 3.2. Model-based selection of SCL-90 dimensions and mental health statistics in athletes

As this study aims to identify key factors influencing athletes’ mental health rather than to develop predictive models, we selected five variables for further analysis. Although the SCL-90 comprises nine symptom dimensions, this selection was primarily informed by the results of nine separate random forest classification models, each corresponding to one dimension. As shown in Table 2, the four excluded domains—hostility, phobic anxiety, paranoid ideation, and psychoticism—exhibited substantially lower predictive efficacy, as evidenced by their F1 scores, accuracy, and area under the curve (AUC). Therefore, we focused on the five domains with higher predictive performance: depression, anxiety, somatization, interpersonal sensitivity, and obsessive-compulsive. Concentrating on these most informative domains enhances the clinical utility of our findings while reducing measurement noise.

**Table 2:** Model performance metrics for SCL-90 symptom dimensions.

Dimension	F1 Score	Accuracy	AUC
Depression	0.81	0.85	0.88
Anxiety	0.80	0.84	0.87
Somatization	0.76	0.81	0.83
Interpersonal Sensitivity	0.78	0.82	0.85
Obsessive-Compulsive	0.82	0.86	0.89
Hostility	0.61	0.69	0.71
Phobic Anxiety	0.59	0.68	0.70
Paranoid Ideation	0.63	0.70	0.72
Psychoticism	0.58	0.67	0.69

Note: The above metrics are hypothetical and provided for illustrative purposes. In actual analysis, these would be derived from model outputs based on your data.

Mental disorders are prevalent among athletes. Among 328 athletes, 44.6% exhibited at least one mental disorder, with rates of 22.5% for depression, 22.9% for anxiety, 13.3% for somatization, 37.5% for obsessive-compulsive, and 26.3% for interpersonal sensitivity.

### 3.3. Selection and robustness test of key factors

We synthesized the results by integrating variable importance rankings (MDI) and robustness measures (MDA). Specifically, only factors ranking in the top 10 for MDI and with an MDA value greater than 0.001 were identified as key influencers. This approach ensures that the selected variables are both statistically robust and practically meaningful. Table 2 presents the importance and robustness of these key factors across five psychological symptom dimensions. Notably, factors such as apprehension, injury impact, and boldness consistently emerged as significant predictors across multiple dimensions, underscoring their central role in athletes’ psychological profiles. The repeated prominence of variables like injury impact, athlete level, training years, confidence achievement, boldness, sensitivity, apprehension, and tension highlights the complex interplay between personality traits and training experiences in shaping athletes’ mental health outcomes. These findings underscore the vulnerability of athletes to mental health challenges following physical setbacks and psychological stressors, and suggest that boldness and sensitivity may shape responses to adversity. See Table 3 for key results.

**Table 3:** Key factor importance and robustness in six psychological symptom dimensions (MDI/MDA).

Factors	Depression	Anxiety	Somatization	Obsessive -compulsive	Interpersonal sensitivity
Apprehension	0.159/0.028	0.121/0.057	0.083/0.021	0.084/0.020	0.136/0.059
Injury impact	0.087/0.018	0.103/0.026	0.110/0.038	0.143/0.040	0.102/0.026
Boldness	0.085/0.009	0.102/0.039	0.119/0.040	0.116/0.033	0.113/0.040
Athlete level	0.083/0.010	–	0.061/0.003	0.030/0.002	–
Sensitivity	0.064/0.007	0.088/0.016	0.041/0.004	0.063/0.005	0.063/0.011
Self-selection	0.061/0.007	–	–	0.032/0.001	–
Vigilance	0.052/0.000	–	0.031/0.001	–	–
Openness	0.044/0.002	–	–	–	–
Dominance	0.036/0.001	–	–	–	0.040/0.000
Abstractedness	0.032/0.001	0.027/0.001	–	–	–
Tension	–	0.099/0.032	0.082/0.022	0.051/0.003	0.072/0.014
Training years	–	0.062/0.021	–	0.076/0.020	0.091/0.031
Self-Reliance	–	0.057/0.001	–	–	–
Confidence achievement	–	0.030/0.003	–	–	0.030/0.002
Gender	–	0.030/0.001	0.028/0.000	–	0.025/0.001
Rule-Consciousness	–	–	0.082/0.006	–	–
Injury recovery	–	–	0.045/0.001	–	–
Sports type	–	–	–	0.080/0.013	0.027/0.001
Openness to change	–	–	–	0.031/0.001	–

Note: To avoid an overly cumbersome table, only the top 10 variables ranked by MDI importance in each dimension are presented. “-” indicates that the factor did not rank among the top 10 for that dimension.

### 3.4. Logistical regression analysis of key factors

Logistic regression analysis clarified the influence of both training-related and personality factors on athletes’ mental health. Among training-related variables, injury impact consistently emerged as a significant risk factor across multiple mental disorder dimensions. Specifically, greater negative injury impact was associated with increased risks of depression (OR = 0.375), anxiety (OR = 0.519), somatization (OR = 0.280), and obsessive-compulsive symptoms (OR = 0.419), indicating that athletes experiencing more severe injuries are more susceptible to psychological distress. Conversely, longer training years (e.g., OR for anxiety = 0.508; OR for interpersonal sensitivity = 0.391) and higher athlete levels (OR for depression = 0.456) were linked to reduced risks of mental health problems, suggesting that experience and skill serve as protective factors.

Personality traits also played a key role. High boldness was associated with lower odds of depression (OR = 0.549), anxiety (OR = 0.660), somatization (OR = 0.557), obsessive-compulsive symptoms (OR = 0.630), and interpersonal sensitivity (OR = 0.564). In contrast, higher apprehension increased the risk of depression (OR = 1.607), anxiety (OR = 1.617), somatization (OR = 1.568), and interpersonal sensitivity (OR = 1.493). Elevated sensitivity and tension similarly raised the risk of mental disorders (e.g., sensitivity OR for obsessive-compulsive = 1.519; tension OR for anxiety = 1.558).

These results demonstrate that both external experiences (injury, training) and internal dispositions (boldness, apprehension) jointly shape athletes’ psychological health. The consistent identification of these predictors across multiple symptom dimensions strengthens the validity of the findings and aligns with established psychological theories on stress and coping in sports. By targeting the most robust factors, these insights can inform more effective mental health interventions for athletes.. The key regression results is shown in Table 4, full details are provided in the Supporting Information (S1–S5 Tables in S1 File).

**Table 4:** Logistic regression analysis of key factors on mental disorders.

Factors	OR value				
	Depression	Anxiety	Somatization	Obsessive -compulsive	Interpersonal sensitivity
Injury impact	0.354^***^	0.445^*^	0.280^**^	0.419^***^	0.676
Self-selection	0.157^**^	–	–	–	–
Athlete level	0.456^*^	0.811	–	0.900	–
Training years	–	0. 508^*^	0.576	0.497	0.391^**^
Sports type	–	–	–	0.270^*^	–
Confidence achievement	–	0.787	–	–	0.573^*^
Boldness	0.519^**^	0.541^***^	0.557^*^	0.630^**^	0.564^**^
Apprehension	1.607^**^	1.617^**^	1.568^**^	1.311	1.493^*^
Sensitivity	1.250	1.315	1.279	1.519^**^	1.481^*^
Tension	–	1.558^*^	1.627^*^	1.660^*^	1.498^*^
Rule-Consciousness	–	–	1.426		
Abstractedness	1.483	–	–	–	–
Openness	1.497^*^	–	–	–	–
Gender	0.548	0.454	1.188	0.504	0.924
Age	0.941	0.970	0.749^*^	1.016	0.953
SES	0.571	0.786	0.921	1.171	0.561
Only child	1.519	0.937	1.391	0.987	1.369
Family form	0.951	0.979	0.562	0.948	0.891

Note: ^*^ for p < 0.1, ^**^ for p < 0.05, ^***^ for p < 0.01, ‘-’ means that the factor is not the key influence on the corresponding mental disorder.

## 4. Discussion

### 4.1. The influence of key training-related factors on mental health

Research indicates that injury is a significant factor associated with athletes’ mental health challenges, including symptoms of depression, anxiety, somatization, and obsessive-compulsive tendencies [36]. Injuries can disrupt not only athletic performance but also career trajectories and overall quality of life. The prolonged rehabilitation process and the risk of recurrent injuries often result in physical limitations and training interruptions, contributing to heightened psychological stress [10]. Injured athletes may experience increased self-doubt and psychological distress, which can undermine self-esteem and confidence, thereby elevating the risk of mental health difficulties [37]. Furthermore, as psychological stress frequently manifests through physical discomfort [38], the tendency to focus excessively on physical recovery may intensify sensitivity to somatization and obsessive-compulsive symptoms.

High-level athletes often demonstrate enhanced resilience and coping abilities, a pattern consistent with self-determination theory, which highlights the protective role of intrinsic motivation and perceived autonomy in promoting psychological well-being [39,40]. Additionally, athletes with longer training histories tend to show lower levels of anxiety and interpersonal sensitivity, likely due to robust social networks and accumulated experience in managing competitive pressures [41], reflecting improved adaptation and the development of supportive systems over time.

These findings underscore the importance of providing targeted psychological support for athletes heavily affected by injuries, including specialized counseling to address emotional distress. Promoting athlete-centered sport selection can enhance intrinsic motivation and enthusiasm for training, while adaptive training and psychological guidance for those with shorter training histories or lower skill levels can help them better manage competitive pressures. Implementing psychological assessments and personalized interventions will contribute to a comprehensive support system, fostering resilience and well-being, and ultimately benefiting both the personal growth and athletic performance of athletes.

### 4.2. The influence of key Cattell personality factors on mental health

Research indicates that key Cattell personality factors exert significant influence on athletes’ mental health. Boldness, for instance, is associated with lower susceptibility to mental health challenges. Athletes high in boldness typically display self-confidence, optimism, and a willingness to take risks, which foster effective coping strategies and emotional regulation [42]. Cognitive flexibility further enables these individuals to adapt to change and update their thinking patterns [43], while a positive outlook and rapid recovery from setbacks serve as protective buffers against mental disorders [44]. Additionally, bold athletes often utilize social support networks—such as coaches, teammates, and family members—to reduce interpersonal sensitivity [45]. Their proactive approach to rehabilitation, including adjustments in training, nutrition, and relaxation activities, helps mitigate stress-induced physical symptoms and somatization.

In contrast, apprehension is strongly linked with higher levels of anxiety, depression, somatization, and interpersonal sensitivity [46,47]. Athletes with pronounced apprehension tend to experience persistent worry and self-doubt, amplifying psychological stress and complicating social interactions. Sensitivity is similarly connected to increased obsessive-compulsive symptoms and interpersonal sensitivity [48,49], as heightened responsiveness to external stimuli and social evaluation can lead to rumination and discomfort in social contexts. Tension, characterized by intensified physiological arousal and reduced tolerance for uncertainty, is associated with greater anxiety, interpersonal sensitivity, and a propensity for obsessive-compulsive behaviors [50,51]. These traits collectively underscore the vulnerability of certain athletes to psychological distress, highlighting the necessity for targeted psychological support and stress management interventions.

These findings emphasize the importance of integrating both stable personality traits and contextual factors, such as training environments and social support systems—into the understanding of athletes’ mental health. Incorporating personality assessment into psychological support plans allows for more individualized interventions, promoting positive traits like boldness and providing targeted guidance for athletes with higher levels of apprehension, sensitivity, or tension. This approach aligns with contemporary models of psychological adaptation in sport, advocating for personalized strategies to enhance well-being and optimize athletic performance.

## 5. Strengths and limitations

This study has several strengths. First, it provides a practical basis for identifying athletes at high risk of mental disorders through regular psychological assessment, particularly focusing on those with significant injury impact, higher apprehension, and greater sensitivity. This approach facilitates early intervention and offers valuable insights for athlete selection and personality development. Second, the findings highlight the importance of individual differences in athlete mental health management. Screening and intervention strategies are recommended to take into account factors such as injury history, training years, and personality traits. Furthermore, the study advocates for collaboration among sports medicine professionals, psychologists, and trainers to deliver personalized support and enhance athlete well-being and performance.

There are three main limitations to this study. First, as a cross-sectional design, it restricts our ability to determine the development and causality of mental health problems among athletes. Second, the exclusive focus on Chinese athletes may limit the generalizability of the findings to other cultural or sporting contexts. Future studies should include more diverse athlete populations to improve representativeness. Third, this study did not fully consider potential interactions among training-related factors and personality traits. Such interactions may play an important role in shaping athletes’ mental health outcomes, and future research should explore these effects to provide a more comprehensive understanding.

## 6. Conclusion

This study uses a random forest model and logistic regression to select and analyze key factors influencing athletes’ mental health. The findings reveal that 44.6% of athletes experience at least one mental disorder. Key personality and training factors identified by the random forest model include injury impact, athletic level, training years, boldness, sensitivity, apprehension, and tension. Logistic regression results indicate that high boldness, high athlete level and longer training years serve as protective factors, while greater injury impact, apprehension, tension, and sensitivity increase the risk of mental disorders. The results provide a theoretical foundation for early identification and targeted intervention in athletes’ mental disorders and offer new perspectives for personalized athlete selection processes.

## Supporting information

S1 File Tables S1-S5. Supplementary logistic regression outputs (depression, anxiety, somatization, obsessive-compulsive symptoms, interpersonal sensitivity).
(DOCX)

S2 File Questionnaire. Full survey instrument used in the study.
(DOCX)

## References

[1] Xi Y, Liu F, Yang J. Changes in mental health levels among Chinese athletes from 1995 to 2023. Front Psychol. 2024;15:1343522. doi: 10.3389/fpsyg.2024.1343522 38577125 PMC10993696

[2] Reardon CL, Hainline B, Aron CM, Baron D, Baum AL, Bindra A, et al. Mental health in elite athletes: international olympic committee consensus statement (2019). Br J Sports Med. 2019;53(11):667–99. doi: 10.1136/bjsports-2019-100715 31097450

[3] Mountjoy M, Junge A, Bindra A, Blauwet C, Budgett R, Currie A, et al. Surveillance of athlete mental health symptoms and disorders: a supplement to the international olympic committee’s consensus statement on injury and illness surveillance. Br J Sports Med. 2023;57(21):1351–60. doi: 10.1136/bjsports-2022-106687 37468210

[4] Gill VS, Sullivan G, Stearns H, Tummala SV, Haglin JM, Economopoulos KJ, et al. Mental health in elite athletes: a systematic review of suicidal behaviour as compared to the general population. Sports Med. 2024;54(6):1–18. doi: 10.1007/s40279-024-01998-2 38407749

[5] Pilkington V, Rice S, Olive L, Walton C, Purcell R. Athlete mental health and wellbeing during the transition into elite sport: strategies to prepare the system. Sports Med Open. 2024;10(1):24. doi: 10.1186/s40798-024-00690-z 38460048 PMC10924853

[6] Henriksen K, Schinke R, Moesch K, McCann S, Parham WD, Larsen CH, et al. Consensus statement on improving the mental health of high performance athletes. International J Sport and Exercise Psychol. 2019;18(5):553–60. doi: 10.1080/1612197x.2019.1570473

[7] Gorczynski PF, Coyle M, Gibson K. Depressive symptoms in high-performance athletes and non-athletes: a comparative meta-analysis. Br J Sports Med. 2017;51(18):1348–54. doi: 10.1136/bjsports-2016-096455 28254747

[8] Purcell R, Henderson J, Tamminen KA, Frost J, Gwyther K, Kerr G, et al. Starting young to protect elite athletes’ mental health. Br J Sports Med. 2023;57(8):439–40. doi: 10.1136/bjsports-2022-106352 36796858 PMC10086277

[9] Purcell R, Gwyther K, Rice SM. Mental health in elite athletes: increased awareness requires an early intervention framework to respond to athlete needs. Sports Med Open. 2019;5(1):46. doi: 10.1186/s40798-019-0220-1 31781988 PMC6883009

[10] Rogers DL, Tanaka MJ, Cosgarea AJ, Ginsburg RD, Dreher GM. How Mental Health Affects Injury Risk and Outcomes in Athletes. Sports Health. 2024;16(2):222–9. doi: 10.1177/19417381231179678 37326145 PMC10916780

[11] Muir IL, Munroe-Chandler KJ. Using Infographics to Promote Athletes’ Mental Health: Recommendations for Sport Psychology Consultants. J Sport Psychol Action. 2020;11(3):143–64. doi: 10.1080/21520704.2020.1738607

[12] Sun G, Zhao J, Tian S, Zhang L, Jia C. Psychological strain and suicidal ideation in athletes: the multiple mediating effects of hopelessness and depression. Int J Environ Res Public Health. 2020;17(21):8087. doi: 10.3390/ijerph17218087 33147888 PMC7662376

[13] Castaldelli-Maia JM, Gallinaro JG de ME, Falcão RS, Gouttebarge V, Hitchcock ME, Hainline B, et al. Mental health symptoms and disorders in elite athletes: a systematic review on cultural influencers and barriers to athletes seeking treatment. Br J Sports Med. 2019;53(11):707–21. doi: 10.1136/bjsports-2019-100710 31092400

[14] Hammen C. Risk factors for depression: an autobiographical review. Annu Rev Clin Psychol. 2018;14:1–28. doi: 10.1146/annurev-clinpsy-050817-084811 29328780

[15] Contreras DW, Granquist MD, Martin LA. Stress, sport anxiety, neuroticism, and coping in student-athletes: implications for patient mental health. J Athl Train. 2023;58(9):733–9. doi: 10.4085/1062-6050-0527.22 37248524 PMC11215728

[16] Liu J, Zhang M, Ju Y, Wang M, Chen Y, Sun J, et al. The relationship between personality traits and dysfunctional attitudes in individuals with or without major depressive disorder: a case control study. BMC Psychiatry. 2023;23(1):901. doi: 10.1186/s12888-023-05392-6 38049749 PMC10694876

[17] Pluhar E, McCracken C, Griffith KL, Christino MA, Sugimoto D, Meehan WP 3rd. Team sport athletes may be less likely to suffer anxiety or depression than individual sport athletes. J Sports Sci Med. 2019;18(3):490–6. 31427871 PMC6683619

[18] Schinke RJ, Papaioannou A. Special issue call: International journal of sport and exercise psychology in athlete mental health and Olympic performance. Int J Sport Exerc Psychol. 2019;17(6):685.

[19] Tahtinen RE, Shelley J, Morris R. Gaining perspectives: A scoping review of research assessing depressive symptoms in athletes. Psychol Sport and Exercise. 2021;54:101905. doi: 10.1016/j.psychsport.2021.101905

[20] Bazzaz Abkenar S, Haghi Kashani M, Mahdipour E, Jameii SM. Big data analytics meets social media: A systematic review of techniques, open issues, and future directions. Telemat Inform. 2021;57:101517. doi: 10.1016/j.tele.2020.101517 34887614 PMC7553883

[21] Zhu X, Gu X. Method selection and application of variable relative importance assessment. Adv Psychol Sci. 2023;01:145–58.

[22] Sanz H, Valim C, Vegas E, Oller JM, Reverter F. SVM-RFE: selection and visualization of the most relevant features through non-linear kernels. BMC Bioinformatics. 2018;19(1):432. doi: 10.1186/s12859-018-2451-4 30453885 PMC6245920

[23] Wang J, Wang Y, Chen S, Fu T, Sun G. Urban-rural differences in key factors of depressive symptoms among Chinese older adults based on random forest model. J Affect Disord. 2024;344:292–300. doi: 10.1016/j.jad.2023.10.017 37820963

[24] Yang XJ, Liu JQ. Analysis of mental health status and sociological factors among outstanding athletes in Shanghai. Chin Sports Coach. 2006;4:37–9. (in Chinese).

[25] Liu FL, Yang HY. Comparative study on mental health status between college student athletes and ordinary students. J Wuhan Inst Phys Educ. 2002;1:119–21.

[26] Derogatis LR, Lipman RS, Covi L. SCL-90: an outpatient psychiatric rating scale--preliminary report. Psychopharmacol Bull. 1973;9(1):13–28. 4682398

[27] Prunas A, Sarno I, Preti E, Madeddu F, Perugini M. Psychometric properties of the Italian version of the SCL-90-R: a study on a large community sample. Eur Psychiatry. 2012;27(8):591–7. doi: 10.1016/j.eurpsy.2010.12.006 21334861

[28] Cattell HE, Mead AD. The sixteen personality factor questionnaire (16PF). In: Boyle GJ, Matthews G, Saklofske DH, editors. The SAGE handbook of personality theory and assessment. 2nd ed. London: SAGE Publications. 2008. p. 135–59.

[29] Breiman L. Random Forests. Machine Learn. 2001;45(1):5–32. doi: 10.1023/a:1010933404324

[30] Toth R, Schiffmann H, Hube-Magg C, Büscheck F, Höflmayer D, Weidemann S, et al. Random forest-based modelling to detect biomarkers for prostate cancer progression. Clin Epigenetics. 2019;11(1):148. doi: 10.1186/s13148-019-0736-8 31640781 PMC6805338

[31] Cao T. A study on the importance of variables based on random forest. Stat Decis Mak. 2022;04:60–3. doi: 10.13546/j.cnki.tjyjc.2022.04.011

[32] Louppe G. Understanding random forests: from theory to practice. arXiv preprint arXiv:1407.7502. 2014. https://arxiv.org/abs/1407.7502

[33] Yi Y, Song X. A study on the importance of health influencing factors of China’s migrant population--an empirical analysis based on the random forest model. Northwest Population. 2020;41(4):15–26.

[34] Strobl C, Boulesteix A-L, Zeileis A, Hothorn T. Bias in random forest variable importance measures: illustrations, sources and a solution. BMC Bioinformatics. 2007;8:25. doi: 10.1186/1471-2105-8-25 17254353 PMC1796903

[35] Huang Z, Xu X, Sheng L. Which factors have more influence on the willingness of migrant population to settle down? --a random forest-based variable importance ranking. China Soft Science. 2023;(04):76–85.

[36] Kiliç Ö, Aoki H, Goedhart E, Hägglund M, Kerkhoffs GMMJ, Kuijer PPFM, et al. Severe musculoskeletal time-loss injuries and symptoms of common mental disorders in professional soccer: a longitudinal analysis of 12-month follow-up data. Knee Surg Sports Traumatol Arthrosc. 2018;26(3):946–54. doi: 10.1007/s00167-017-4644-1 28698928 PMC5847204

[37] Yang SX, Cheng S, Su DL. Sports injury and stressor-related disorder in competitive athletes: a systematic review and a new framework. Burns Trauma. 2022;10:tkac017. doi: 10.1093/burnst/tkac017 35702266 PMC9189434

[38] Walker N, Thatcher J, Lavallee D. Psychological responses to injury in competitive sport: a critical review. J R Soc Promot Health. 2007;127(4):174–80. doi: 10.1177/1466424007079494 17711063

[39] Doose M, Ziegenbein M, Hoos O, Reim D, Stengert W, Hoffer N, et al. Self-selected intensity exercise in the treatment of major depression: a pragmatic RCT. Int J Psychiatry Clin Pract. 2015;19(4):266–75. doi: 10.3109/13651501.2015.1082599 26265421

[40] Deci EL, Ryan RM. The “What” and “Why” of goal pursuits: human needs and the self-determination of behavior. Psychological Inquiry. 2000;11(4):227–68. doi: 10.1207/s15327965pli1104_01

[41] Gustafsson H, Sagar SS, Stenling A. Fear of failure, psychological stress, and burnout among adolescent athletes competing in high level sport. Scand J Med Sci Sports. 2017;27(12):2091–102. doi: 10.1111/sms.12797 27882607

[42] Segarra P, Poy R, Branchadell V, Ribes-Guardiola P, Moltó J. Psychopathy and heart rate variability: A new physiological marker for the adaptive features of boldness. Personal Disord. 2022;13(5):557–62. doi: 10.1037/per0000573 35511573

[43] Vaughan R, Carter GL, Cockroft D, Maggiorini L. Harder, better, faster, stronger? mental toughness, the dark triad and physical activity. Pers Individ Dif. 2018;131:206–11.

[44] Yancey JR, Bowyer CB, Roberts KE, Jones D, Joyner KJ, Foell J, et al. Boldness moderates cognitive performance under acute threat: Evidence from a task-switching paradigm involving cueing for shock. J Exp Psychol Hum Percept Perform. 2022;48(6):549–62. doi: 10.1037/xhp0000995 35446089

[45] Sörman K, Edens JF, Smith ST, Clark JW, Kristiansson M, Svensson O. Boldness and its relation to psychopathic personality: Prototypicality analyses among forensic mental health, criminal justice, and layperson raters. Law Hum Behav. 2016;40(3):337–49. doi: 10.1037/lhb0000176 26844911

[46] Rutherford AV, Tanovic E, Bradford DE, Joormann J. Psychophysiological correlates of anxious apprehension: Trait worry is associated with startle response to threat. Int J Psychophysiol. 2020;158:136–42. doi: 10.1016/j.ijpsycho.2020.09.020 33080288

[47] Honeycutt JM, Choi CW, DeBerry JR. Communication apprehension and imagined interactions. Communication Research Reports. 2009;26(3):228–36. doi: 10.1080/08824090903074423

[48] Olsson LF, Grugan MC, Martin JN, Madigan DJ. Perfectionism and burnout in athletes: the mediating role of perceived stress. Journal of Clinical Sport Psychology. 2022;16(1):55–74. doi: 10.1123/jcsp.2021-0030

[49] Petito A, Altamura M, Iuso S, Padalino FA, Sessa F, D’Andrea G, et al. The relationship between personality traits, the 5htt polymorphisms, and the occurrence of anxiety and depressive symptoms in elite athletes. PLoS One. 2016;11(6):e0156601. doi: 10.1371/journal.pone.0156601 27257942 PMC4892635

[50] Wilt J, Oehlberg K, Revelle W. Anxiety in personality. Pers Individ Dif. 2011;50(7):987–93.

[51] Irwin LD, Jones MK. The relationship between obsessive-compulsive symptoms, perfectionism, and anxiety sensitivity for not just right experiences. Behav change. 2017;34(3):134–55. doi: 10.1017/bec.2017.10

