# Supplementary material for: Key personality and training factors influencing athletes’ mental health - based on machine learning
Source: PLoS One. 2025 Dec 17;20(12):e0335918. doi: 10.1371/journal.pone.0335918 (PMC12711052; doi:10.1371/journal.pone.0335918)
Supplement: S1 File — Supplementary logistic regression outputs (depression, anxiety, somatization, obsessive-compulsive symptoms, interpersonal sensitivity). (DOCX) [file pone.0335918.s001.docx]

# Table S1-S5

Table S1 Logistic regression analysis of key factors (Depression)

| Factors | reference | B | OR | 95%CI |
| --- | --- | --- | --- | --- |
| Injury impact | Very bad | -0.953 | 0.354^***^ | (0.212，0.662) |
| Self-selection | No | -1.852 | 0.157^**^ | (0.039，0.634) |
| Athlete level | Other=1 | -0.721 | 0.456^*^ | (0.265，0.893) |
| Boldness |  | -0.580 | 0.519^**^ | (0.374，0.804) |
| Abstractedness |  | 0.394 | 1.483 | (0.956，2.300) |
| Apprehension |  | 0.437 | 1.607^**^ | (1.179, 2.244) |
| Openness to Change |  | 0.404 | 1.497^*^ | (1.005，2.232) |
| Sensitivity |  | 0.223 | 1.250 | (0.921，1.697) |
| Gender | Female | -0.602 | 0.548 | (0.214, 1.401) |
| Age |  | -0.061 | 0.941 | (0.796, 1.112) |
| SES | Poor | -0.560 | 0.571 | (0.324, 1.008) |
| Only child | No | 0.418 | 1.519 | (0.589, 3.917) |
| Family form | Other | -0.050 | 0.951 | (0.325, 2.789) |

Note: ^*^ for p < 0.1, ^**^ for p < 0.05, ^***^ for p < 0.01.

Table S2 Logistic regression analysis of key factors (Anxiety)

| Factors | reference | B | OR | 95%CI |
| --- | --- | --- | --- | --- |
| Athlete level | Other | -0.209 | 0.811 | (0.584，1.126) |
| Injury impact | Very bad | -0.778 | 0.445^**^ | (0.285，0.726) |
| Confidence achievement | Very bad | -0.239 | 0.787 | (0.458，1.353) |
| Training years | 0-5 | -0.676 | 0.508^*^ | (0.280，0.924) |
| Boldness |  | -0.614 | 0.541^**^ | (0.377，0.778) |
| Sensitivity |  | 0.274 | 1.315 | (0.952，1.1817) |
| Abstractedness |  | 0.481 | 1.617^**^ | (1.127，2.320) |
| Tension |  | 0.444 | 1.558^*^ | (1.054，2.304) |
| Gender | Female | -0.791 | 0.454 | (0.163，1.258) |
| Age |  | -0.031 | 0.970 | (0.803，1.117) |
| SES | Poor | -0.241 | 0.786 | (0.431，1.435) |
| Only child | No | 0.905 | 0.937 | (0.324，2.712) |
| Family form | Other | 0.973 | 0.979 | (0.289，3.232) |

Note: ^*^ for p < 0.1, ^**^ for p < 0.05, ^***^ for p < 0.01.

Table S3 Logistic regression analysis of key factors (Somatization)

| Factors | reference | B | OR | 95%CI |
| --- | --- | --- | --- | --- |
| Injury impact | Very bad | -1.274 | 0.280^**^ | (0.106，0.738) |
| Training years | 0-5 | -0.552 | 0.576 | (0.607，1.663) |
| Rule-Consciousness |  | 0.355 | 1.426 | (0.801，2.537) |
| Boldness |  | -0.560 | 0.557^*^ | (0.336，0.972) |
| Abstractedness |  | 0.450 | 1.568^**^ | (1.118，2.199) |
| Sensitivity |  | 0.246 | 1.279 | (0.886，1.847) |
| Tension |  | 0.487 | 1.627^*^ | (1.103，2.399) |
| Gender | Female | 0.173 | 1.188 | (0.404, 3.495) |
| Age |  | -0.289 | 0.749^*^ | (0.595, 0.943) |
| SES | Poor | -0.082 | 0.921 | (0.450, 1.884) |
| Only child | No | 0.330 | 1.391 | (0.418, 4.628) |
| Family form | Other | -0.575 | 0.562 | (0.157, 2.012) |

Note: ^*^ for p < 0.1, ^**^ for p < 0.05, ^***^ for p < 0.01.

Table S4 Logistic regression analysis of key factors (Obsessive-compulsive)

| Factors | reference | B | OR | 95%CI |  |
| --- | --- | --- | --- | --- | --- |
| Injury impact | Very bad | -0.869 | 0.419^***^ | (0.255，0.689) |  |
| Athlete level | | Other | 0.796 | 0.900 | (0.672，1.207) |
| Sports type | Individual | -1.308 | 0.270^*^ | (0.073，0.999) |  |
| Training years | 0-5 | -0.699 | 0.497 | (0.237，1.041) |  |
| Abstractedness |  | 0.271 | 1.311 | (0.939，1.829) |  |
| Boldness |  | -0.462 | 0.630^**^ | (0.473，0.841) |  |
| Sensitivity |  | 0.418 | 1.519^**^ | (1.110，2.077) |  |
| Tension |  | 0.507 | 1.660* | (1.093，2.522) |  |
| Gender | Female | -0.684 | 0.504 | (0.195, 1.304) |  |
| Age |  | 0.015 | 1.016 | (0.862, 1.196) |  |
| SES | Poor | 0.158 | 1.171 | (0.643, 2.134) |  |
| Only child | No | -0.013 | 0.987 | (0.361, 2.697) |  |
| Family form | Other | -0.054 | 0.948 | (0.326, 2.759) |  |

Note: ^*^ for p < 0.1, ^**^ for p < 0.05, ^***^ for p < 0.01

Table S5 Logistic regression analysis of key factors (Interpersonal sensitivity)

| Factors | reference | B | OR | 95%CI |
| --- | --- | --- | --- | --- |
| Injury impact | Very bad | -0.392 | 0.676 | (0.439，1.040) |
| Training years | 0-5 | -0.940 | 0.391^**^ | (0.215，0.710) |
| Confidence achievement | Very bad | -0.556 | 0.573^*^ | (0.344，0.956) |
| Boldness |  | -0.573 | 0.564^**^ | (0.383，0.831) |
| Abstractedness |  | 0.401 | 1.493^*^ | (1.048，2.128) |
| Sensitivity |  | 0.393 | 1.481^*^ | (1.063，2.063) |
| Tension |  | 0.404 | 1.498^*^ | (1.035，2.167) |
| Gender | Female | -0.079 | 0.924 | (0.373, 2.290) |
| Age |  | -0.048 | 0.953 | (0.802, 1.132) |
| SES | Poor | -0.577 | 0.561 | (0.312, 1.010) |
| Only child | No | 0.314 | 1.369 | (0.518, 3.621) |
| Family form | Other | -0.116 | 0.891 | (0.294, 2.702) |

Note: ^*^ for p < 0.1, ^**^ for p < 0.05, ^***^ for p < 0.01

# Ethical Approval

| 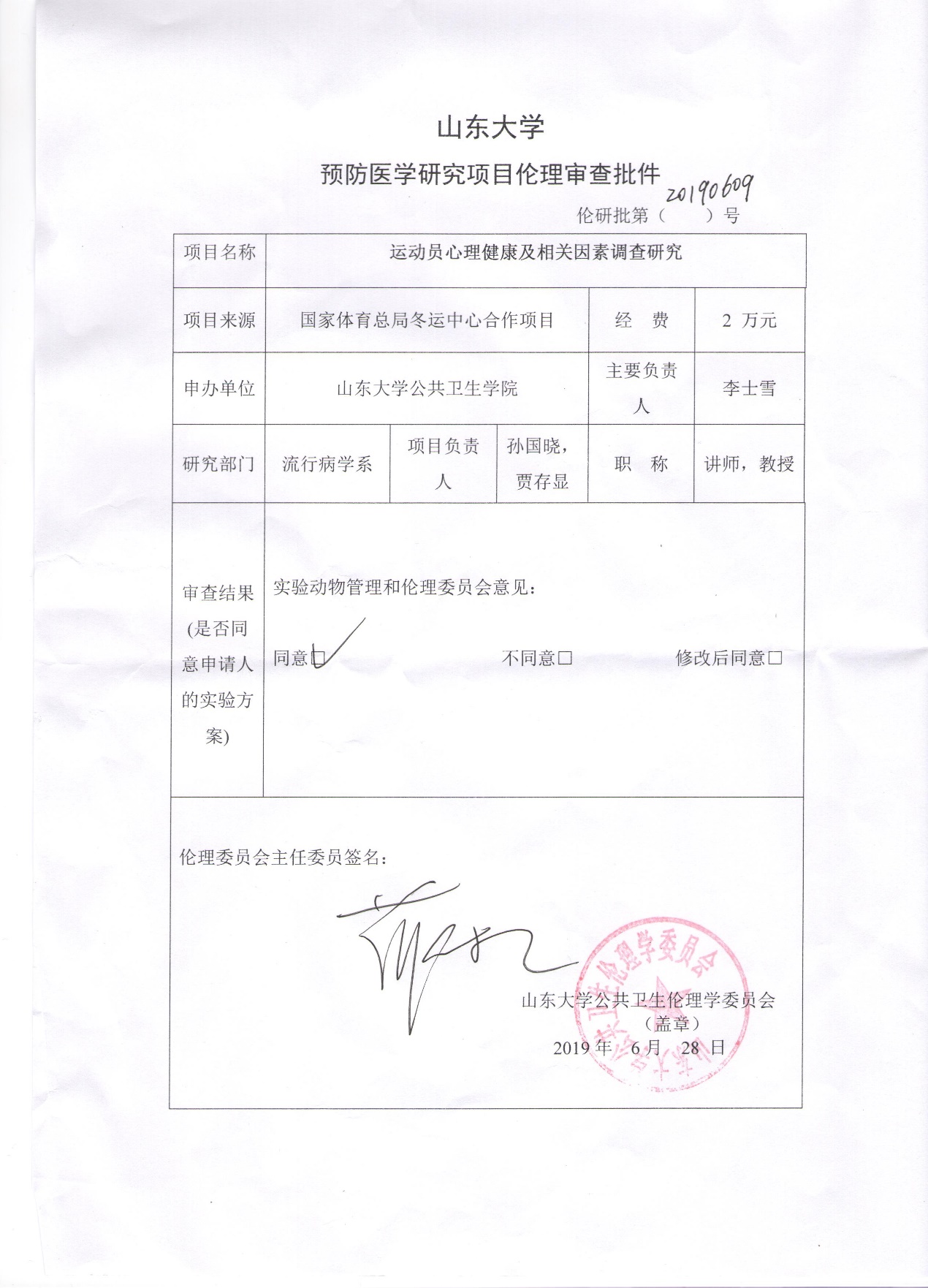 |
| --- |

| Project Title | Survey Study on Athletes' Mental Health and Related Factors | | | | |
| --- | --- | --- | --- | --- | --- |
| Source of Project | Cooperative Project with the Winter Sports Center of the General Administration of Sport | | | Funding | 20,000 |
| Applicant Institution | School of Public Health, Shandong University | | | Main Responsible Person | Li ShiXue |
| Research Department | Department of Epidemiology | Project Leader | Sun Guoxiao, Jia CunXian | Professional Title | Lecturer, Professor |
| Review Outcome (Approval of the Applicant's Experimental Plan) | the Animal Ethics and Welfare Committee agrees with the experimental plan.  Agree Disagree Agree with modifications | | | | |
| Signature of the Chairperson of the Ethics Committee:  Shandong University Public Health Ethics Committee (Seal)  Date: June 28, 2019 | | | | | |

**Shandong University**

**Ethical Approval for Preventive Medicine Research Project**

Ethical Approval Number: (20190609)

Date of Approval: 20190609
