# Supplementary material for: Key personality and training factors influencing athletes’ mental health - based on machine learning
Source: PLoS One. 2025 Dec 17;20(12):e0335918. doi: 10.1371/journal.pone.0335918 (PMC12711052; doi:10.1371/journal.pone.0335918)
Supplement: S2 File — Full survey instrument used in the study. (DOCX) [file pone.0335918.s002.docx]

运动员基本信息情况表（心理普查专用）

**一、个人信息**

姓名：_____________ 年龄： __________ 性别：_____________

出生年月：____________ 出生地：_________________（省、市）

**二、运动信息**

1. 当前从事具体运动项目名称：_______________
2. 当前从事运动项目级别：（如公斤级，没有可填无）______________
3. 当前主教练或执教教练：____________________
4. 参与所有专业运动训练总的年限（四舍五入为整年）：___________

5、最近一年内是否存在更换运动项目或项目级别： A.是 B.否

6、进入山东省体育训练中心前是否有在体校专业体育训练经历：

A.是 B.否

7、最近一年运动损伤对你运动表现的影响：A.非常重大 B.比较重大

C.中等 D.比较小 E.非常小

8、最近一年你对运动损伤的恢复预期：A.非常差 B.比较差 C.中等

D.比较好 E.非常好

1. 对自己当前从事的运动专项，获取成就的信心：A.非常大 B.比较大 C.中等 D.比较小 E.非常小

10、目前从事的运动专项是否由自己选择：A.是 B.否

**三、家庭信息**

1. 生长家庭的形态（0-18岁时的主要家庭形态）：A.一般双亲家庭 B.离异单亲家庭 C.疾病意外等导致的单亲家庭 D.重组家庭 E.三代及以上共同生活家庭 F其他组织形式家庭
2. 自我评价家庭经济状况：A.优越 B.良好 C.一般 D.较差 E.其他

3、出生顺序：A.独生子女 B.长子女 C.次子女 D.第三位及以上

4、家庭对你目前从事运动的支持程度：A.非常大 B.比较大 C.中等

D.比较小 E.非常小

5、在训练中遇到困难是否愿意与家人沟通：A.是 B.否

6、每周与父母沟通的频率：A.一周五次或以上 B.一周三次

C.一周一次 D.一周零次

7、与家庭沟通中多数关于哪方面：A.运动效果 B.生活状态 C.人际关系 D.其他____________（请写出）

**四、寄宿生活背景信息**

1. 寄宿生活开始于几岁：A. 6岁以下 B. 6—12岁 C. 12—16岁 D. 16岁以上 E.未开始寄宿生活
2. 寄宿生活累计至今已有多久：A. 2年以内 B. 2—4年 C. 4—8年 D. 8年以上 E.没有寄宿生活经历

**五、其他生活信息**

1、你目前生活最大的乐趣是：__________________________________

2、你记忆中最深刻的一件事情是什么？请写出：

___________________________________________________________3、当自己运动效果不佳时，你常采用什么办法突破这种状况？请写出：________________________________________________________

**《卡特尔十六种人格因素测验》测试题目**

1、我很明了本测验的说明：

是的 B不一定 C不是的

2、我对本测验每一小问题都会按自己的真实情况作答：

A是的 B不一定 C不同意

3、有度假机会时，我宁愿：

A去一个繁华的都市 B介于A与C之间 C闲居清静而偏僻的郊区

4、我有足够的能力应付困难：

A是的 B不一定 C不是的

5、即使是见到关在铁笼内的猛兽也会使我惴惴不安：

A是的 B不一定 C不是的

6、我总避免批评别人的言行：

A是的 B有时如此 C不是的

7、我的思想似乎：

A走在时代的前面 B不一定 C正符合时代

8、我不擅长说笑话和讲趣事：

A是的 B介于A与C之间 C不是的

9、当我看到亲友邻居争执时，我总是：

A任其自己解决 B置之不理 C予以解决

10、在社交场合中，我：

A谈吐自然 B介于A与C之间 C退避三舍，保持沉默

11、我愿做一名：

A建筑工程师 B不确定 C社会科学的教员

12、阅读时，我宁愿选读：

A著名的宗教教义 B不确定 C国家政治组织的理论

13、我相信许多人都有些心理不正常，虽然他们都不愿意承认：

A是的 B介于A与C之间 C不是的

14、我所希望的结婚对象应擅长交际而无需有文艺才能：

A是的 B不一定 C不是的

15、对于头脑简单和不讲理的人，我仍然能待之以礼：

A是的 B介于A与C之间 C不是的

16、受人侍奉时我常感到不安：

A是的 B介于A与C之间 C不是的

17、从事体力或脑力劳动后，我比平常人需要更多的休息：

A是的 B介于A与C之间 C不是的

18、半夜醒来，我会为种种忧虑而不能入眠：

A常常如此 B有时如此 C极少如此

19、事情进行不顺利时，我会急得掉眼泪：

A从不如此 B有时如此 C时常如此

20、我认为只要双方同意就可以离婚，不应当受传统礼教的束缚：

A是的 B介于A与C之间 C不是的

21、我对于人或物的兴趣很容易改变：

A是的 B介于A与C之间 C不是的

22、筹划事务时，我宁愿：

A和别人合作 B不确定 C自己单独进行

23、我常会无端地自言自语：

A常常如此 B偶然如此 C从不如此

24、无论工作、饮食或出游，我总：

A很匆忙，不能尽兴 B介于A与C之间 C很从容不迫

25、有时我会怀疑别人是否真正地对我的言谈有兴趣：

A是的 B介于A与C之间 C不是的

26、在工厂中，我宁愿负责：

A机械组 B介于A与C之间 C人事组

27、在阅读时，我宁愿选读：

A太空旅行 B不太确定 C家庭教育

28、下列三个字中哪个字与其它两个字属于不同类别：

A狗 B石 C牛

29、如果我能重新做人，我要：

A把生活安排得和以前不同 B不确定 C生活得和以前相仿

30、在我一生之中，我总能达到我预期的目标：

A是的 B不一定 C不是的

31、当我说谎时，我总觉得内心不安，不敢正视对方：

A是的 B不一定 C不是的

32、假如我手持一支装有子弹的手枪，我必须取出子弹后才能安心：

A是的 B介于A与C之间 C不是的

33、朋友们大都认为我是一个说话很风趣的人：

A是的 B不一定 C不是的

34、如果人们知道我的内心世界，他们都会感到惊讶：

A是的 B不一定 C不是的

35、在社交场合中，如果我突然成为众所注意的中心，我会感到局促不安：

A是的 B介于A与C之间 C不是的

36、我总喜欢参加规模庞大的聚会、舞会或公共集会：

A是的 B介于A与C之间 C不是的

37、在下列工作中，我喜欢的是：

A音乐 B不一定 C手工

38、我常常怀疑那些过于友善的人动机是否如此：

A是的 B介于A与C之间 C不是的

39、我宁愿自己的生活象：

A一个艺人或博物学家 B不确定 C会计师或保险公司的经纪人

40、目前世界所需要的是：

A富有改善世界计划的理想家 B不确定 C脚踏实地的可靠公民

41、有时我觉得我需要做剧烈的体力活动：

A是的 B介于A与C之间 C不是的

42、我愿意与有礼貌有教养的人来往，而不愿和粗鲁野蛮的人为伍：

A是的 B介于A与C之间 C不是的

43、在处理一些必须凭借智慧的事务中，我的父母的确：

A较一般人差 B普通 C超人一等

44、当上司（或教师）召见我时，我：

A总觉得可以趁机提出建议 B介于A与C之间 C总怀疑自己做错了什么事

45、假使薪奉优厚，我愿意专任照料精神病人的职务：

A是的 B介于A与C之间 C不是的

46、看报时，我喜欢读：

A当前世界基本问题的辩论 B介于A与C之间 C地方新闻的报道

47、我曾担任过：

A一种职务 B多种职务 C非常多的职务

48、逛街时我宁愿观看一个画家写生，而不愿听人家的辩论：

A是的 B不一定 C不是的

49、我的神经脆弱，稍有刺激的声音就会使我颤惊：

A时常如此 B有时如此 C从未如此

50、我在清早起身时，常常感到疲乏不堪：

A是的 B介于A与C之间 C不是的

51、我宁愿是一个：

A森林的工作人员 B不一定 C中小学教员

52、每逢过节或亲人生日，我：

A喜欢互相赠送礼物 B不太确定 C觉得交换礼物是件麻烦事

53、下列数字中，哪个数字与其它两个数字属于不同类别：

A 5 B 2 C 7

54、“猫”与“鱼”就如同“牛”与：

A牛乳 B牧草 C盐

55、在做人处世的各个方面，我的父母很令人敬佩：

A是的 B不一定 C不是的

56、我觉得我有一些别人所不及的优良品质：

A是的 B不一定 C不是的

57、只要有利于大家，尽管是别人认为卑贱的工作，我也乐而为之，不以为耻：

A是的 B不太确定 C不是的

58、我喜欢看电影或参加其他娱乐活动：

A每周一次以上（比一般人多） B每周一次（与通常人相似） C偶然一次（比通常人少）

59、我喜欢从事需要更精确技术的工作：

A是的 B介于A与C之间 C不是的

60、在有理想、有地位的长者面前，我总较为缄默：

A是的 B介于A与C之间 C不是的

61、对我来说，在大众面前表演或演讲是一件不容易的事：

A是的 B介于A与C之间 C不是的

62、我宁愿：

A指挥几个人工作 B不确定 C和团体共同工作

63、纵使我做了一桩贻笑大方的事，我也仍然能够将它淡然忘却：

A是的 B介于A与C之间 C不是的

64、没有人会幸灾乐祸地希望我遭遇困难：

A是的 B不确定 C不是的

65、堂堂男子汉应该：

A考虑人生的意义 B不确定 C谋家庭的温饱

66、我喜欢解决别人已弄得一塌糊涂的问题：

A是的 B介于A与C之间 C不是的

67、我十分高兴的时候总有好景不长之感：

A是的 B介于A与C之间 C不是的

68、在一般的困难处境下，我总能保持乐观

A是的 B不一定 C不是的

69、迁居是一件极不愉快的事

A是的 B介于A与C之间 C不是的

70、在我年轻的时候，如果我和父母的意见不同，我经常：

A坚持自己的意见 B介于A与C之间 C接受他们的意见

71、我希望我的爱人能够使家庭

A有其本身的欢乐与活动 B介于A与C之间 C成为邻里社交活动的一部分

72、我解决问题多数依靠：

A个人独立思考 B介于A与C之间 C与人互相讨论

73、需要当机立断时，我总：

A镇静地运用理智 B介于A与C之间 C常常紧张兴奋，不能冷静思考

74、最近，在一两桩事上，我觉得自己是无辜受累：

A是的 B介于A与C之间 C不是的

75、我善于控制我的表情：

A是的 B介于A与C之间 C不是的

76、如果薪奉相等，我宁愿做：

A一个化学研究师 B不确定 C旅行社经理

77、“惊讶”与“新奇”尤如“惧怕”与：

A勇敢 B焦虑 C恐怖

78、下列三个分数中，哪一个与其他两个属不同类别

A 3/7 B 3/9 C 3/11

79、不知什么缘故，有些人故意回避或冷落我：

A是的 B不一定 C不是的

80、我虽善意待人，却得不到好报：

A是的 B不一定 C不是的

81、我不喜欢那些夜郎自大，目空一切的人：

A是的 B介于A与C之间 C不是的

82、和一般人相比，我的朋友的确太少：

A是的 B介于A与C之间 C不是的

83、万不得已时我才参加社交集会，否则我总是设法回避：

A是的 B不一定 C不是的

84、在服务机关中，对上级的逢迎得当，比工作上的表现更为重要：

A是的 B介于A与C之间 C不是的

85、参加竞赛活动时，我看重的是竞赛活动，而不计较其成败：

A总是如此 B一般如此 C偶然如此

86、我宁愿我所从事的职业有：

A固定可靠的薪水 B介于A与C之间 C新资高低能随我工作的表现而随时调整

87、我宁愿阅读：

A军事与政治的事实记载 B不一定 C一部富有情感与幻想的作品

88、有许多人不干欺骗犯罪，主要原因是怕受到惩罚

A是的 B介于A与C之间 C不是的

89、我的父母（或监护人）从未很严格地要我事事顺从：

A是的 B不一定 C不是的

90、“百折不挠”、“再接再厉”的精神似乎完全被现代人忽视了：

A是的 B不一定 C不是的

91、如果有人对我发怒，我总：

A设法使他镇静下来 B不太确定 C也会恼怒起来

92、我希望大家都提倡：

A多吃蔬菜以避免杀生 B不一定 C发展农业，扑灭对农产品有害的动物

93、无论在极高的屋顶上还是极深的隧道中，我很少觉得胆怯不安：

A是的 B介于A与C之间 C不是的

94、我只要没有过错，不管人家怎样归咎于我，我总能心安理得：

A是的 B不一定 C不是的

95、凡是无法运用理智来解决的问题，有时就不得不靠权力来处理：

A是的 B介于A与C之间 C不是的

96、我十六、七岁时与异性朋友的交游：

A极多 B介于A与C之间 C比别人冷淡

97、我是交际场合或所参加的组织中的一个活跃分子：

A是的 B介于A与C之间 C不是的

98、在人声噪杂中，我仍能不受妨碍、专心工作：

A是的 B介于A与C之间 C不是的

99、在某些心境下，我常因困惑引起幻想而将工作搁置下来

A是的 B介于A与C之间 C不是的

100、我很少用令人难堪的话去伤别人的感情：

A是的 B不太确定 C不是的

101、我更愿意做一名：

A商店经理 B不确定 C建筑师

102、“理不胜辞”的意思是：

A理不如辞 B理多而辞寡 C辞藻丰富而理由不足

103、“锄头”与“挖掘”尤如“刀子”与

A雕刻 B切剖 C铲除

104、我常横过街道，以回避我不愿与之打招呼的人

A很少如此 B偶然如此 C有时如此

105、在我倾听音乐时，如果人家高谈阔论：

A我仍能够专心听，不受影响 B介于A与C之间 C我会因不能专心欣赏而感到恼怒

106、在课堂上，如果我的意见与老师不同，我常：

A保持缄默 B不一定 C当场表明立场

107、我和异性友伴交谈时，极力避免有关“性”的话题：

A是的 B介于A与C之间 C不是的

108、我待人接物的确不太成功：

A是的 B不尽然 C不是的

109、每当考虑困难问题时，我总是：

A一切都未雨绸缪 B介于A与C之间 C相信到时候会自然解决

110、我所结交的朋友中男女各占一半：

A是的 B介于A与C之间 C不是的

111、我宁可：

A结识很多的人 B不一定 C维持几个深交的朋友

112、我宁为哲学家，而不作机械工程师：

A是的 B不确定 C不是的

113、如果我发现某人自私不义时，我总不计一切指责他的弱点：

A是的 B介于A与C之间 C不是的

114、我善用心机去影响同伴，使他们能协调实现我的目标：

A是的 B介于A与C之间 C不是的

115、我喜欢做戏剧、音乐、歌剧等新闻采访工作：

A是的 B不一定 C不是的

116、当人们颂扬我时，我总觉得不好意思：

A是的 B介于A与C之间 C不是的

117、我认为现代最需要解决的问题是：

A政治纠纷 B不太确定 C道德目标的有无

118、我有时会无故地产生一种面临横祸的恐惧：

A是的 B有时如此 C不是的

119、我在童年时，害怕黑暗的次数：

A极多 B不太多 C没有

120、黄昏闲暇，我喜欢：

A看一部历史探险片 B不一定 C看一本科学幻想小说

121、当人们批评我古怪时，我觉得：

A非常气恼 B有些动气 C无所谓

122、在一个陌生的城市找住址时，我经常：

A向人问路 B介于A与C之间 C参考市区地图

123、朋友申言要在家休息时，我会仍设法怂恿他们外出：

A是的 B不一定 C不是的

124、在就寝时，我：

A不易入睡 B介于A与C之间 C极容易入睡

125、有人烦扰我时，我：

A能不露声色 B介于A与C之间 C要说给别人听，以泄气愤

126、如果薪奉相等，我宁愿做一个：

A律师 B不确定 C飞行员或航海员

127、时间永恒是比喻：

A时间过得很慢 B忘了时间 C光阴一去不复返

128、下列三项记号中，哪一项应紧接：×○○○○××○○○×××

A ×○× B ○○× C ○××

129、在陌生的地方，我仍能清楚地辨别东西南北各方向：

A是的 B介于A与C之间 C不是的

130、我的确比一般人幸运，因为我能从事自己所喜欢的工作：

A是的 B不一定 C不是的

131、如果我急于想借用别人的东西而物主恰又不在，我认为不告而取也无大碍：

A是的 B介于A与C之间 C不是的

132、我喜欢向友人追述一些以往有趣的社交经验：

A是的 B介于A与C之间 C不是的

133、我更愿意做一名：

A演员 B不确定 C建筑师

134、工作学习之余，我总要安排计划，不使时间浪费：

A是的 B介于A与C之间 C不是的

135、与人交际时，我常会无端地产生一种自卑感：

A是的 B介于A与C之间 C不是的

136、主动与陌生人交谈：

A是一件难事 B介于A与C之间 C毫无困难

137、我喜欢的音乐多数是：

A轻快活泼 B介于A与C之间 C富于情感

138、我爱做“白日梦”即“完全沉浸于幻想之中”：

A是的 B不一定 C不是的

139、未来20年的世界局势定将好转：

A是的 B不一定 C不是的

140、童年时我喜欢阅读：

A战争故事 B不确定 C神仙幻想故事

141、我素来对机械、汽车、飞机等有兴趣：

A是的 B介于A与C之间 C不是的

142、我愿意做一个缓刑释放罪犯的管理监视人：

A是的 B介于A与C之间 C不是的

143、人们认为我不过是一个能苦干，稍有成就的人而已：

A是的 B介于A与C之间 C不是的

144、在逆境中，我总能保持精神振奋：

A是的 B介于A与C之间 C不是的

145、我认为人工节育是解决世界经济、和平问题的要诀：

A是的 B不太确定 C不是的

146、我喜欢独自筹划，避免别人的干涉和建议：

A是的 B介于A与C之间 C不是的

147、我相信“上司不可能没有过错，但他仍有权做当权者”：

A是的 B不一定 C不是的

148、我总设法使自己不粗心大意，忽略细节：

A是的 B介于A与C之间 C不是的

149、与人争辩或险遭事故后，我常发抖、精疲力竭，不能安心工作：

A是的 B介于A与C之间 C不是的

150、没有医生处方，我从不乱用药：

A是的 B介于A与C之间 C不是的

151、为了培养个人兴趣，我愿意参加：

A摄影组 B不确定 C辩论会

152、“星火燎原”对等于“姑息：

A同情” B养奸” C纵容”

153、“钟表”与“时间”尤如“裁缝”与

A西装 B剪刀 C布料

154、生动的梦境常常滋扰我的睡眠：

A时常如此 B偶然如此 C从未如此

155、我过去曾撕毁一些禁止人们自由的布告：

A是的 B介于A与C之间 C不是的

156、在一个陌生的城市中，我会：

A到处游荡 B不确定 C避免去较不安全的地方

157、我宁愿服饰素洁大方，而不愿争奇斗艳惹人注目：

A是的 B不太确定 C不是的

158、黄昏时安静的娱乐远胜过热闹的宴会：

A是的 B不太确定 C不是的

159、我常常明知故犯，不愿意接受好心的建议：

A偶然如此 B罕有如此 C从不如此

160、我总把“是非”、“善恶”作为判断或取舍的原则：

A是的 B介于A与C之间 C不是的

161、我工作时不喜欢有许多人在场参观：

A是的 B介于A与C之间 C不是的

162、我故意为难一些有教养的人，如医生、教师，这是一件有趣的事：

A是的 B介于A与C之间 C不是的

163、在各种课程中，我较喜欢：

A语文 B不确定 C数学

164、那些自以为是，道貌岸然的人最使我生气：

A是的 B介于A与C之间 C不是的

165、与平常循规蹈矩的人交谈：

A颇有兴趣，亦有所得 B介于A与C之间 C他们思想肤浅使我厌烦

166、我喜欢：

A有几个对我很苛刻，但富有感情的朋友 B介于A与C之间 C不受别人的牵涉

167、如果作民意投票时，我宁愿投票赞同：

A切实根绝有心理缺陷者的生育 B不确定 C对杀人犯判处死刑

168、我有时会无端地感到沮丧痛苦：

A是的 B介于A与C之间 C不是的

169、当我与立场相反的人辩论时，我主张：

A尽量找出基本观点的差异 B不一定 C彼此让步以解决矛盾

170、我一向重感情而不重理智，因此我的观点常常动摇不定：

A是的 B大致如此 C不是的

171、我的学习效率有赖于：

A阅读好书 B介于A与C之间 C参加团体讨论

172、我宁愿选一个高薪的工作，不在乎有无保障；而不愿任低薪的固定工作：

A是的 B不太确定 C不是的

173、在参加辩论以前，我总先把握住自己的立场：

A经常如此 B一般如此 C必要时才如此

174、我常被一些无谓的琐事所烦扰：

A是的 B介于A与C之间 C不是的

175、我宁愿住在嘈杂的城市，而不愿住在安静的乡村：

A是的 B不太确定 C不是的

176、我宁愿：

A负责领导儿童游戏 B不确定 C协助钟表修理

177、一人一事，众人受累，我对这句话的反应是：

A愤 B偾 C喷

178、望子成龙的家长，往往（）苗助长：

A揠 B堰 C偃

179、气候的转变并不影响我的情绪：

A是的 B介于A与C之间 C不是的

180、因为我对于一切问题都有些见解，大家公认我富于思想：

A是的 B介于A与C之间 C不是的

181、我讲话的声音：

A洪亮 B介于A与C之间 C低沉

182、人们公认我是一个活跃热情的人：

A是的 B介于A与C之间 C不是的

183、我喜欢有旅行和变动机会的工作，而不计较工作本身之是否有保障：

A是的 B介于A与C之间 C不是的

184、我治事严格，凡事都务求正确尽善：

A是的 B介于A与C之间 C不是的

185、在取回或归还东西时，我总仔细检查东西是否还保持原状：

A是的 B介于A与C之间 C不是的

186、我通常精力充沛，忙碌多事：

A是的 B不一定 C不是的

187、我确信我没有遗漏或不经心回答上面任何问题：

A是的 B不确定 C不是的

**《心理健康临床症状自评量表》测试题目**

1. 头痛

A、没有　B、很轻　C、中等　D、偏重　E、严重

1. 神经过敏，心中不踏实

A、没有　B、很轻　C、中等　D、偏重　E、严重

1. 头脑中有不必要的想法或字句盘旋

A、没有　B、很轻　C、中等　D、偏重　E、严重

1. 头昏或昏倒

A、没有　B、很轻　C、中等　D、偏重　E、严重

1. 对异性的兴趣减退

A、没有　B、很轻　C、中等　D、偏重　E、严重

1. 对旁人求全责备

A、没有　B、很轻　C、中等　D、偏重　E、严重

1. 感到别人能控制你的思想

A、没有　B、很轻　C、中等　D、偏重　E、严重

1. 责怪别人制造麻烦

A、没有　B、很轻　C、中等　D、偏重　E、严重

1. 忘性大

A、没有　B、很轻　C、中等　D、偏重　E、严重

1. 担心自己的衣饰整齐及仪态的端正

A、没有　B、很轻　C、中等　D、偏重　E、严重

1. 容易烦恼和激动

A、没有　B、很轻　C、中等　D、偏重　E、严重

1. 脚痛

A、没有　B、很轻　C、中等　D、偏重　E、严重

1. 害怕空旷的场所或街道

A、没有　B、很轻　C、中等　D、偏重　E、严重

1. 感到自己的精力下降，活动减慢

A、没有　B、很轻　C、中等　D、偏重　E、严重

1. 想结束自己的生命

A、没有　B、很轻　C、中等　D、偏重　E、严重

1. 听到旁人听不到的声音

A、没有　B、很轻　C、中等　D、偏重　E、严重

1. 发抖

A、没有　B、很轻　C、中等　D、偏重　E、严重

1. 感到大多数人都不可信任

A、没有　B、很轻　C、中等　D、偏重　E、严重

1. 胃口不好

A、没有　B、很轻　C、中等　D、偏重　E、严重

1. 容易哭泣

A、没有　B、很轻　C、中等　D、偏重　E、严重

1. 同异性相处时感到害羞不自在

A、没有　B、很轻　C、中等　D、偏重　E、严重

1. 感到受骗，中了圈套或有人想住自己

A、没有　B、很轻　C、中等　D、偏重　E、严重

1. 无缘无故地突然感到害怕

A、没有　B、很轻　C、中等　D、偏重　E、严重

1. 自己不能控制地大发脾气

A、没有　B、很轻　C、中等　D、偏重　E、严重

1. 怕单独出门

A、没有　B、很轻　C、中等　D、偏重　E、严重

1. 经常责怪自己

A、没有　B、很轻　C、中等　D、偏重　E、严重

1. 腰痛

A、没有　B、很轻　C、中等　D、偏重　E、严重

1. 感到难以完成任务

A、没有　B、很轻　C、中等　D、偏重　E、严重

1. 感到孤独

A、没有　B、很轻　C、中等　D、偏重　E、严重

1. 感到苦闷

A、没有　B、很轻　C、中等　D、偏重　E、严重

1. 过分担忧

A、没有　B、很轻　C、中等　D、偏重　E、严重

1. 对事物不感兴趣

A、没有　B、很轻　C、中等　D、偏重　E、严重

1. 感到害怕

A、没有　B、很轻　C、中等　D、偏重　E、严重

1. 我的感情容易受到伤害

A、没有　B、很轻　C、中等　D、偏重　E、严重

1. 旁人能知道我的私下想法

A、没有　B、很轻　C、中等　D、偏重　E、严重

1. 感到别人不理解我或不同情我

A、没有　B、很轻　C、中等　D、偏重　E、严重

1. 感到别人对我不友好，不喜欢我

A、没有　B、很轻　C、中等　D、偏重　E、严重

1. 做事必须做得很慢以保证做得准确

A、没有　B、很轻　C、中等　D、偏重　E、严重

1. 心跳得很厉害

A、没有　B、很轻　C、中等　D、偏重　E、严重

1. 恶心或胃部不舒服

A、没有　B、很轻　C、中等　D、偏重　E、严重

1. 感到比不上他人

A、没有　B、很轻　C、中等　D、偏重　E、严重

1. 肌肉酸痛

A、没有　B、很轻　C、中等　D、偏重　E、严重

1. 感到有人在监视我谈论我

A、没有　B、很轻　C、中等　D、偏重　E、严重

1. 难以入睡

A、没有　B、很轻　C、中等　D、偏重　E、严重

1. 做事必须反复检查

A、没有　B、很轻　C、中等　D、偏重　E、严重

1. 难以做出决定

A、没有　B、很轻　C、中等　D、偏重　E、严重

1. 怕乘电车、公共汽车、地铁或火车

A、没有　B、很轻　C、中等　D、偏重　E、严重

1. 呼吸有困难

A、没有　B、很轻　C、中等　D、偏重　E、严重

1. 一阵阵发冷或发热

A、没有　B、很轻　C、中等　D、偏重　E、严重

1. 因为感到害怕而避开某些东西、场合或活动

A、没有　B、很轻　C、中等　D、偏重　E、严重

1. 脑子变空了

A、没有　B、很轻　C、中等　D、偏重　E、严重

1. 身体发麻或刺痛

A、没有　B、很轻　C、中等　D、偏重　E、严重

1. 喉咙有梗塞感

A、没有　B、很轻　C、中等　D、偏重　E、严重

1. 感到前途没希望

A、没有　B、很轻　C、中等　D、偏重　E、严重

1. 不能集中注意力

A、没有　B、很轻　C、中等　D、偏重　E、严重

1. 感到身体某一部分软弱无力

A、没有　B、很轻　C、中等　D、偏重　E、严重

1. 感到紧张或容易紧张

A、没有　B、很轻　C、中等　D、偏重　E、严重

1. 感到手或脚发重

A、没有　B、很轻　C、中等　D、偏重　E、严重

1. 想到死亡的事

A、没有　B、很轻　C、中等　D、偏重　E、严重

1. 吃得太多

A、没有　B、很轻　C、中等　D、偏重　E、严重

1. 当别人看着我或谈论我时感到不自在

A、没有　B、很轻　C、中等　D、偏重　E、严重

1. 有一些不属于你自己的想法

A、没有　B、很轻　C、中等　D、偏重　E、严重

1. 有想打人或伤害他人的冲动

A、没有　B、很轻　C、中等　D、偏重　E、严重

1. 醒得太早

A、没有　B、很轻　C、中等　D、偏重　E、严重

1. 必须反复洗手、点数目或触摸某些东西

A、没有　B、很轻　C、中等　D、偏重　E、严重

1. 睡得不稳不深

A、没有　B、很轻　C、中等　D、偏重　E、严重

1. 有想摔坏或破坏东西的冲动

A、没有　B、很轻　C、中等　D、偏重　E、严重

1. 有一些别人没有的想法或念头

A、没有　B、很轻　C、中等　D、偏重　E、严重

1. 感到对别人神经过敏

A、没有　B、很轻　C、中等　D、偏重　E、严重

1. 在商店或电影院等人多的地方感到不自在

A、没有　B、很轻　C、中等　D、偏重　E、严重

1. 感到任何事情都很困难

A、没有　B、很轻　C、中等　D、偏重　E、严重

1. 一阵阵恐惧或惊恐

A、没有　B、很轻　C、中等　D、偏重　E、严重

1. 感到在公共场合吃东西很不舒服

A、没有　B、很轻　C、中等　D、偏重　E、严重

1. 经常与人争论

A、没有　B、很轻　C、中等　D、偏重　E、严重

1. 单独一人时神经很紧张

A、没有　B、很轻　C、中等　D、偏重　E、严重

1. 别人对我的成绩没有作出恰当的评价

A、没有　B、很轻　C、中等　D、偏重　E、严重

1. 即使和别人在一起也感到孤单

A、没有　B、很轻　C、中等　D、偏重　E、严重

1. 感到坐立不安心神不定

A、没有　B、很轻　C、中等　D、偏重　E、严重

1. 感到自己没有什么价值

A、没有　B、很轻　C、中等　D、偏重　E、严重

1. 感到熟悉的东西变得陌生或不像是真的

A、没有　B、很轻　C、中等　D、偏重　E、严重

1. 大叫或摔东西

A、没有　B、很轻　C、中等　D、偏重　E、严重

1. 害怕会在公共场合昏倒

A、没有　B、很轻　C、中等　D、偏重　E、严重

1. 感到别人想占我的便宜

A、没有　B、很轻　C、中等　D、偏重　E、严重

1. 为一些有关“性”的想法而很苦恼

A、没有　B、很轻　C、中等　D、偏重　E、严重

1. 我认为应该因为自己的过错而受到惩罚

A、没有　B、很轻　C、中等　D、偏重　E、严重

1. 感到要赶快把事情做完

A、没有　B、很轻　C、中等　D、偏重　E、严重

1. 感到自己的身体有严重问题

A、没有　B、很轻　C、中等　D、偏重　E、严重

1. 从未感到和其他人很亲近

A、没有　B、很轻　C、中等　D、偏重　E、严重

1. 感到自己有罪

A、没有　B、很轻　C、中等　D、偏重　E、严重

1. 感到自己的脑子有毛病

A、没有　B、很轻　C、中等　D、偏重　E、严重

Basic Information Table of Athletes (for Psychological Census)

**1、 Personal Information**

Name: __________ Age: __________ Gender:_____________

Date of Birth: __________ Place of Birth: _________________ (Province, City)

**2、 Sports information**

1. Current specific sports project name:_______________
2. Current level of sports activity: (e.g. kilogram level, none to be filled in)______________
3. Current head coach or coaching coach:____________________
4. Total years of participation in all professional sports training (rounded to the nearest whole year):___________

5. Have there been any changes in sports events or event levels in the past year? A. Yes B. No

6. Have you had professional sports training experience at a sports school before entering the Shandong Provincial Sports Training Center

A. Yes B. No

7. The impact of sports injuries on your athletic performance in the past year: A Very significant B. Relatively significant

C. Medium D. relatively small E. very small

8. Your expectation for recovery from sports injuries in the past year: A Very poor B. relatively poor C. moderate

D. Quite good E. very good

1. Confidence in achieving success in one's current sports field: A Very large B. relatively large C. moderate D. relatively small E. very small

10. Is the current sports specialty chosen by oneself? A Yes B. No

**3、 Family Information**

1. Form of Growing Family (Main Family Form at 0-18 Years Old): A General two parent families B. Divorced single parent families C. Single parent families caused by illness, accidents, etc. D. Reorganized families E. Families with three or more generations living together F Other organizational forms of families
2. Self evaluation of family economic status: A Superior B. Good C. General D. Poor E. Other

3. Birth order: A Only child B. eldest child C. second child D. third child or above

4. The level of support from your family for your current involvement in sports: A Very large B. relatively large C. moderate

D. Relatively small E. very small

5. Are you willing to communicate with your family when encountering difficulties during training? A Yes B. No

6. Frequency of weekly communication with parents: A Five or more times a week B. Three times a week

C. Once a week D. Zero times a week

7. What aspects are most discussed in communication with families: A Sports effects B. Lifestyle status C. Interpersonal relationships D. Other __________ (please write)

**4、 Background information on boarding life**

1. Boarding life begins at what age: A Under 6 years old B. 6-12 years old C. 12-16 years old D. Over 16 years old E. Not started boarding life
2. How long has the boarding life accumulated so far?: A Within 2 years B. 2-4 years C. 4-8 years D. 8 years or more E. No residential living experience

**5、 Other life information**

1. The greatest joy in your current life is:__________________________________

2. What is the most memorable thing in your memory? Please write:

___________________________________________________________3. What methods do you often use to overcome poor exercise results? Please write:________________________________________________________

**Test Questions for the Sixteen Personality Factors of the Cartel Test (16PF)**

1. I am well aware of the instructions for this test:

Yes, B doesn't necessarily mean C. It's not true

2. I will answer every small question in this test based on my own real situation:

A Yes B Not necessarily C Disagree

3. When there is a vacation opportunity, I would rather:

A goes to a bustling city, B is between A and C, and C lives in a quiet and remote suburb

4. I have enough ability to cope with difficulties:

A is, B may not necessarily be, C is not

5. Even seeing a fierce beast locked in an iron cage makes me uneasy:

A is, B may not necessarily be, C is not

6. I always avoid criticizing others' words and actions:

A is, B sometimes is, C is not

7. My thoughts seem to be:

A is ahead of the times, B may not necessarily be in line with the times, and C is in line with the times

8. I am not good at telling jokes and interesting things:

A is yes, B is between A and C, and C is not

9. When I see family, friends, and neighbors arguing, I always:

A can solve it on its own, B ignores it, and C solves it

10. In social situations, I:

A speaks naturally, B is between A and C, C retreats and remains silent

11. I would like to be a:

A construction engineer B uncertain C social science instructor

12. When reading, I would rather choose to read:

A famous religious doctrine B uncertain C theory of national political organization

13. I believe many people have some psychological abnormalities, although they are unwilling to admit:

A is yes, B is between A and C, and C is not

14. The marriage partner I hope to marry should be good at socializing without needing artistic talent:

A is, B may not necessarily be, C is not

15. I can still treat people with simple minds and irrationality with courtesy:

A is yes, B is between A and C, and C is not

16. I often feel uneasy when being served:

A is yes, B is between A and C, and C is not

17. After engaging in physical or mental labor, I need more rest than an ordinary person:

A is yes, B is between A and C, and C is not

18. Waking up in the middle of the night, I will be unable to sleep due to various worries:

A often does this, B sometimes does this, C rarely does this

19. When things don't go smoothly, I feel so anxious that I shed tears

A is never as good as this. B is sometimes like this. C is often like this

20. I believe that divorce can be done as long as both parties agree, and should not be bound by traditional etiquette:

A is yes, B is between A and C, and C is not

My interest in people or things can easily change:

A is yes, B is between A and C, and C is not

When planning affairs, I would rather:

A collaborates with others, B is uncertain, and C works alone

23. I often talk to myself for no reason:

A often does this, B occasionally does it, C never does it

24. Regardless of work, diet, or travel, I always:

A is in a hurry and cannot fully enjoy themselves. B is between A and C, and C is very calm and composed

Sometimes I wonder if others are truly interested in my words:

A is yes, B is between A and C, and C is not

In the factory, I would rather be responsible for:

A mechanical group B is between A and C, C personnel group

27. When reading, I would rather choose to read:

A space travel B uncertain C family education

28. Which of the following three characters belongs to a different category from the other two characters

A dog B stone C cow

If I could start over, I would:

A arranges life differently from before B is uncertain C lives similarly to before

Throughout my life, I have always been able to achieve my expected goals:

A is, B may not necessarily be, C is not

When I lie, I always feel uneasy inside and dare not face the other person directly:

A is, B may not necessarily be, C is not

32. If I hold a handgun with bullets in my hand, I must remove the bullets before I can feel at ease:

A is yes, B is between A and C, and C is not

Most of my friends think that I am a person who speaks very humorously:

A is, B may not necessarily be, C is not

If people knew my inner world, they would all be surprised:

A is, B may not necessarily be, C is not

35. In social situations, if I suddenly become the center of attention, I will feel uneasy:

A is yes, B is between A and C, and C is not

I always enjoy attending large-scale gatherings, dances, or public gatherings

A is yes, B is between A and C, and C is not

Among the following jobs, which one do I like

A Music B Not necessarily C Handmade

I often doubt whether those overly friendly people have the same motivation:

A is yes, B is between A and C, and C is not

39. I would rather live my life like:

A artist or naturalist B uncertain C accountant or insurance company agent

What the world currently needs is:

A wealthy idealist with plans to improve the world B uncertain C down-to-earth and reliable citizen

Sometimes I feel like I need to do intense physical activity:

A is yes, B is between A and C, and C is not

42. I am willing to associate with polite and cultured people, rather than with rough and barbaric people:

A is yes, B is between A and C, and C is not

In dealing with some matters that require wisdom, my parents do indeed:

A is worse than the average person, B is ordinary, C is superhuman, first class

When my superior (or teacher) summons me, I:

A always feels that they can take the opportunity to make suggestions, B is between A and C, and C always suspects that they have done something wrong

45. If the salary is generous, I am willing to take on the role of caring for mental patients full-time:

A is yes, B is between A and C, and C is not

When reading newspapers, I like to read:

A debate on fundamental global issues B lies between A and C C local news coverage

47. I have served as:

A One Position B Multiple Positions C A Very Many Positions

48. When shopping, I would rather watch a painter sketch than listen to their debate:

A is, B may not necessarily be, C is not

49. My nerves are fragile, and even the slightest sound can make me tremble:

A is always like this, B is sometimes like this, C has never been like this

When I wake up in the early morning, I often feel exhausted:

A is yes, B is between A and C, and C is not

51. I would rather be one:

A forest staff B may not necessarily C primary and secondary school teachers

52. During festivals or family birthdays, I:

A likes to give gifts to each other, B is not sure, C thinks exchanging gifts is a hassle

Which of the following numbers belongs to a different category from the other two numbers

A 5 B 2 C 7

54. "Cat" and "fish" are like "cow" and:

A Milk B Grass C Salt

55. In all aspects of being a person and dealing with people, my parents are very admirable:

A is, B may not necessarily be, C is not

56. I think I have some excellent qualities that others cannot match:

A is, B may not necessarily be, C is not

57. As long as it benefits everyone, even if it is considered lowly by others, I am happy to do it and not ashamed:

A is yes, B is not quite sure, C is not

I enjoy watching movies or participating in other entertainment activities

A More than once a week (more than the average person) B Once a week (similar to the average person) C Occasionally once (less than the average person)

I enjoy working in jobs that require more precise techniques:

A is yes, B is between A and C, and C is not

In front of elders with ideals and status, I always remain silent

A is yes, B is between A and C, and C is not

61. For me, performing or giving a speech in front of the public is not an easy task:

A is yes, B is between A and C, and C is not

62. I would rather:

A commands several people to work, B is uncertain, C works together with the group

Even if I do something ridiculous, I can still forget it calmly:

A is yes, B is between A and C, and C is not

64. No one will gloat and hope that I encounter difficulties:

A is yes, B is uncertain, C is not

65. A true man should:

A considers the meaning of life, B is uncertain, C seeks to provide for their family's basic needs

66. I enjoy solving problems that others have already messed up:

A is yes, B is between A and C, and C is not

67. When I am very happy, I always feel like the good times don't last long:

A is yes, B is between A and C, and C is not

In general difficult situations, I always maintain optimism

A is, B may not necessarily be, C is not

69. Moving is an extremely unpleasant thing

A is yes, B is between A and C, and C is not

When I was young, if I had different opinions from my parents, I often:

A insists on their own opinions, B is between A and C, and C accepts their opinions

71. I hope my lover can help the family

A has its own joy and activities, B is between A and C, and C becomes a part of neighborhood social activities

72. I mostly rely on:

A thinks independently, B is between A and C, and C discusses with others

73. When I need to make a quick decision, I always:

A calmly applies reason, B is between A and C, C is often nervous and excited, unable to think calmly

Recently, in one or two things, I feel innocent and burdened:

A is yes, B is between A and C, and C is not

75. I am good at controlling my facial expressions:

A is yes, B is between A and C, and C is not

If the salary is equal, I would rather do:

A chemical researcher B uncertain C travel agency manager

77. "Surprise" and "novelty" are like "fear" and:

A Brave B Anxious C Terrifying

Which of the following three scores belongs to a different category from the other two

A 3/7 B 3/9 C 3/11

79. For some unknown reason, some people intentionally avoid or ignore me:

A is, B may not necessarily be, C is not

Although I treat others with kindness, I do not receive good rewards

A is, B may not necessarily be, C is not

81. I don't like those night owls who are arrogant and arrogant

A is yes, B is between A and C, and C is not

Compared to ordinary people, I do have too few friends:

A is yes, B is between A and C, and C is not

I only attend social gatherings when absolutely necessary, otherwise I always try to avoid them

A is, B may not necessarily be, C is not

84. In the service mechanism, appropriate flattery towards superiors is more important than performance at work:

A is yes, B is between A and C, and C is not

85. When participating in competition activities, I value the competition activities and do not care about their success or failure:

A is always like this, B is usually like this, C happens to be like this

86. I would rather have the following profession:

A Fixed and Reliable Salary B Between A and C C New salary can be adjusted at any time according to my job performance

87. I would rather read:

A military and political factual record B may not necessarily be C. A work full of emotions and fantasies

88. Many people do not commit fraud crimes mainly because they are afraid of being punished

A is yes, B is between A and C, and C is not

My parents (or guardians) have never strictly required me to obey everything:

A is, B may not necessarily be, C is not

The spirit of "perseverance" and "perseverance" seems to have been completely overlooked by modern people:

A is, B may not necessarily be, C is not

If someone gets angry with me, I always:

A tries to calm him down, B is not sure, C will also get angry

92. I hope everyone advocates for:

A. Eat more vegetables to avoid killing. B. Not necessarily. C. Develop agriculture and eliminate animals harmful to agricultural products

93. Whether on extremely high roofs or in extremely deep tunnels, I rarely feel timid or uneasy:

A is yes, B is between A and C, and C is not

As long as I am not at fault, no matter how others blame me, I can always feel at ease:

A is, B may not necessarily be, C is not

95. Any problem that cannot be solved by reason sometimes has to be dealt with by power:

A is yes, B is between A and C, and C is not

96. My interactions with friends of the opposite sex when I was sixteen or seventeen years old:

A is abundant, B is between A and C, and C is colder than others

97. I am an active member in social situations or organizations I participate in:

A is yes, B is between A and C, and C is not

98. Despite the noise of human voices, I am still able to focus on my work without any hindrance

A is yes, B is between A and C, and C is not

99. In certain states of mind, I often put work on hold due to confusion causing fantasies

A is yes, B is between A and C, and C is not

I rarely use embarrassing words to hurt someone's feelings:

A is yes, B is not quite sure, C is not

101. I would rather be a:

A Store Manager B Uncertain C Architect

102. The meaning of "unreasonable" is:

A reason is not as good as words B. There are too many reasons but few words C. There are too many words but insufficient reasons

103. "hoes" and "digging" are like "knives" and

A carving B cutting C removal

I often cross the street to avoid people I don't want to greet

A rarely does this, B occasionally does it, C sometimes does it

105. When I listen to music, if someone talks loudly:

A: I can still listen attentively without being affected. B: Between A and C. C: I feel angry because I can't concentrate and appreciate

In class, if my opinion differs from the teacher's, I often:

A remains silent B may not necessarily C expresses a position on the spot

When I talk to my opposite sex friends, I try my best to avoid topics related to "sex":

A is yes, B is between A and C, and C is not

108. I am indeed not very successful in dealing with people:

A is yes, B is not entirely true, and C is not

Whenever I consider difficult problems, I always:

A is prepared for everything, B is between A and C, and C believes that it will be resolved naturally when the time comes

110. Half of the friends I have made are men and half are women

A is yes, B is between A and C, and C is not

111. I would rather:

A has met many people, B may not necessarily, C maintains a few close friends

112. I would rather be a philosopher than a mechanical engineer:

A is yes, B is uncertain, C is not

If I find someone selfish and unjust, I always criticize their weaknesses regardless of everything:

A is yes, B is between A and C, and C is not

114. I make good use of my scheming to influence my peers, so that they can coordinate and achieve my goals:

A is yes, B is between A and C, and C is not

I enjoy doing news interviews for dramas, music, operas, and other genres

A is, B may not necessarily be, C is not

When people praise me, I always feel embarrassed:

A is yes, B is between A and C, and C is not

117. In my opinion, the most pressing issue that needs to be addressed in modern times is:

A political dispute B uncertain C existence of moral goals

Sometimes I have an inexplicable fear of facing a disaster:

A is, B sometimes is, C is not

119. The number of times I was afraid of darkness during my childhood:

A is extremely abundant, B is not very abundant, and C is absent

At dusk leisure, I like:

A watching a historical adventure movie B not necessarily C watching a science fiction novel

When people criticize me for being weird, I feel:

A is very angry, B is a bit angry, C doesn't matter

When looking for an address in a strange city, I often:

A asks people for directions, B is between A and C, C refers to the city map

123. When my friends say they want to rest at home, I will still try to encourage them to go out:

A is, B may not necessarily be, C is not

At bedtime, I:

A is difficult to fall asleep, B is between A and C, and C is extremely easy to fall asleep

When someone troubles me, I:

A can remain silent, B is between A and C, and C needs to speak to others to vent anger

If the salary is equal, I would rather do one:

A Lawyer B Uncertain C Pilot or Navigator

127. Eternal time is a metaphor:

A Time flies slowly B Forgetting time C Time is gone forever

128. Which of the following three symbols should be followed immediately

A ×○× B ○○× C ○××

129. In unfamiliar places, I can still clearly distinguish between east, west, north, and south directions:

A is yes, B is between A and C, and C is not

I am indeed luckier than the average person because I am able to pursue a job that I enjoy

A is, B may not necessarily be, C is not

If I am eager to borrow something from someone else and the owner is not there, I think it is not a big deal to take it without informing them:

A is yes, B is between A and C, and C is not

132. I like to recount some interesting social experiences from the past to my friends:

A is yes, B is between A and C, and C is not

133. I would rather be a:

A Actor B Uncertain C Architect

Besides work and study, I always make plans to avoid wasting time

A is yes, B is between A and C, and C is not

135. When interacting with others, I often develop an unfounded sense of inferiority:

A is yes, B is between A and C, and C is not

136. Proactively converse with strangers:

A is a difficult task, B is between A and C, and C is not difficult at all

137. Most of the music I like is:

A is lively and lively, B is between A and C, and C is rich in emotions

138. I love daydreaming, which means being completely immersed in fantasy

A is, B may not necessarily be, C is not

139. The world situation is bound to improve in the next 20 years:

A is, B may not necessarily be, C is not

140. In my childhood, I enjoyed reading:

A War Story B Uncertain C Fairy Fantasy Story

141. I have always been interested in machinery, cars, airplanes, etc

A is yes, B is between A and C, and C is not

142. I am willing to be a management and surveillance officer for released criminals on probation:

A is yes, B is between A and C, and C is not

143. People think that I am just someone who can work hard and achieve some success:

A is yes, B is between A and C, and C is not

144. In adversity, I always keep my spirits high:

A is yes, B is between A and C, and C is not

145. I believe that artificial contraception is the key to solving global economic and peace issues

A is yes, B is not quite sure, C is not

146. I like to plan alone and avoid interference and suggestions from others:

A is yes, B is between A and C, and C is not

I believe that 'a boss cannot be without fault, but he still has the right to be in power':

A is, B may not necessarily be, C is not

I always try my best not to be careless and overlook details:

A is yes, B is between A and C, and C is not

149. After arguing with others or narrowly escaping accidents, I often tremble and feel exhausted, unable to work with peace of mind

A is yes, B is between A and C, and C is not

150. Without a doctor's prescription, I never misuse medication:

A is yes, B is between A and C, and C is not

151. In order to cultivate personal interests, I am willing to participate in:

A Photography Group B Uncertain C Debate Meeting

152. 'Spark ignites the prairie' is equivalent to 'appeasement':

A sympathizes, B nurtures traitors, C condones

153. "Clocks" and "time" are like "tailors" and

A suit B scissors C fabric

154. Vivid dreams often disturb my sleep:

A often does this, B occasionally does this, C has never done this before

155. I have torn up some notices that prohibit people's freedom in the past:

A is yes, B is between A and C, and C is not

In a strange city, I would:

A wanders around, B is uncertain, C avoids going to less safe places

157. I would rather wear simple and elegant clothing than compete for attention:

A is yes, B is not quite sure, C is not

158. Quiet entertainment at dusk far outweighs lively banquets:

A is yes, B is not quite sure, C is not

159. I often knowingly make mistakes and am unwilling to accept well intentioned advice:

A happens to be like this, B rarely is like this, C never is like this

160. I always use "right and wrong" and "good and evil" as principles for judgment or decision-making:

A is yes, B is between A and C, and C is not

161. I don't like having many people present to visit when I'm working:

A is yes, B is between A and C, and C is not

162. I deliberately make things difficult for some educated people, such as doctors and teachers, which is an interesting thing:

A is yes, B is between A and C, and C is not

Among various courses, I prefer:

A Chinese B Uncertain C Mathematics

164. Those self righteous and righteous people make me angry the most:

A is yes, B is between A and C, and C is not

165. Talk to people who usually follow the rules:

A is quite interested and has gained something. B is between A and C. Their shallow thinking makes me annoyed

166. I like:

A has a few friends who are very strict with me, but have strong emotions. B is between A and C. C is not involved by others

167. If conducting a public opinion poll, I would rather vote in favor of:

A. Effectively eradicate the reproduction of individuals with psychological defects B. Uncertain C. Impose the death penalty on murderers

168. Sometimes I feel inexplicably frustrated and painful:

A is yes, B is between A and C, and C is not

When I argue with someone who holds the opposite position, I advocate:

A tries to identify the differences in basic viewpoints as much as possible, B may not necessarily, C compromises with each other to resolve conflicts

170. I have always placed more emphasis on emotions than rationality, so my views often waver:

A is yes, B is roughly like this, C is not

My learning efficiency depends on:

A Reading Good Books B Between A and C C Participating in Group Discussions

172. I would rather choose a high paying job than worry about security; And unwilling to take on low paying fixed jobs:

A is yes, B is not quite sure, C is not

Before participating in debates, I always grasp my own position:

A often does this, B usually does this, C only does it when necessary

174. I am often troubled by meaningless trivialities:

A is yes, B is between A and C, and C is not

I would rather live in a noisy city than a quiet countryside:

A is yes, B is not quite sure, C is not

176. I would rather:

A is responsible for leading children's games, B is uncertain, and C assists in clock repairs

177. One person, one thing, everyone is burdened. My reaction to this sentence is:

A angry B angry C sprayed

178. Parents who aspire for their children to succeed often encourage them to:

A 揠 B Weir C Yan

179. The change in climate does not affect my emotions:

A is yes, B is between A and C, and C is not

180. Because I have some insights into all issues, it is widely recognized that I am rich in ideas:

A is yes, B is between A and C, and C is not

181. The voice of my speech:

A is bright, B is between A and C, and C is low

182. People recognize me as an active and enthusiastic person:

A is yes, B is between A and C, and C is not

183. I enjoy jobs that offer opportunities for travel and change, regardless of whether the job itself is secure

A is yes, B is between A and C, and C is not

184. I am strict in my governance and always strive for correctness and goodness in everything

A is yes, B is between A and C, and C is not

185. When retrieving or returning items, I always carefully check if they are still in their original state:

A is yes, B is between A and C, and C is not

I am usually full of energy and busy with multiple tasks

A is, B may not necessarily be, C is not

187. I am confident that I have not overlooked or answered any of the above questions without careful consideration:

A is yes, B is uncertain, C is not

**Test questions for the Clinical Symptom Self Assessment Scale for Mental Health(SCL-90)**

1. headache

A. No B, very light C, moderate D, heavy E, severe

1. Nervous hypersensitivity, feeling uneasy in the heart

A. No B, very light C, moderate D, heavy E, severe

1. Unnecessary thoughts or words swirling in the mind

A. No B, very light C, moderate D, heavy E, severe

1. Dizziness or fainting

A. No B, very light C, moderate D, heavy E, severe

1. Decreased interest in the opposite sex

A. No B, very light C, moderate D, heavy E, severe

1. Blame others for seeking perfection

A. No B, very light C, moderate D, heavy E, severe

1. Feeling that others can control your thoughts

A. No B, very light C, moderate D, heavy E, severe

1. Blaming others for causing trouble

A. No B, very light C, moderate D, heavy E, severe

1. have a poor memory

A. No B, very light C, moderate D, heavy E, severe

1. Worried about one's neat attire and proper posture

A. No B, very light C, moderate D, heavy E, severe

1. Easy to worry and get excited

A. No B, very light C, moderate D, heavy E, severe

1. Foot pain

A. No B, very light C, moderate D, heavy E, severe

1. Afraid of open spaces or streets

A. No B, very light C, moderate D, heavy E, severe

1. Feeling a decrease in energy and slower activity

A. No B, very light C, moderate D, heavy E, severe

1. I want to end my own life

A. No B, very light C, moderate D, heavy E, severe

1. Hearing sounds that others cannot hear

A. No B, very light C, moderate D, heavy E, severe

1. tremble

A. No B, very light C, moderate D, heavy E, severe

1. Feeling that most people are untrustworthy

A. No B, very light C, moderate D, heavy E, severe

1. Poor appetite

A. No B, very light C, moderate D, heavy E, severe

1. Easy to cry

A. No B, very light C, moderate D, heavy E, severe

1. Feeling shy and uncomfortable when interacting with the opposite sex

A. No B, very light C, moderate D, heavy E, severe

1. Feeling deceived, falling into a trap, or someone trying to hold onto oneself

A. No B, very light C, moderate D, heavy E, severe

1. Suddenly feeling scared for no reason

A. No B, very light C, moderate D, heavy E, severe

1. Uncontrollable outburst of anger

A. No B, very light C, moderate D, heavy E, severe

1. Afraid of going out alone

A. No B, very light C, moderate D, heavy E, severe

1. Frequently blaming oneself

A. No B, very light C, moderate D, heavy E, severe

1. lumbago

A. No B, very light C, moderate D, heavy E, severe

1. Feeling difficult to complete the task

A. No B, very light C, moderate D, heavy E, severe

1. Feeling lonely

A. No B, very light C, moderate D, heavy E, severe

1. Feeling depressed

A. No B, very light C, moderate D, heavy E, severe

1. Excessive worry

A. No B, very light C, moderate D, heavy E, severe

1. Not interested in things

A. No B, very light C, moderate D, heavy E, severe

1. Feeling scared

A. No B, very light C, moderate D, heavy E, severe

1. My emotions are easily hurt

A. No B, very light C, moderate D, heavy E, severe

1. Others can know my private thoughts

A. No B, very light C, moderate D, heavy E, severe

1. Feeling that others do not understand or sympathize with me

A. No B, very light C, moderate D, heavy E, severe

1. I feel that others are unfriendly to me and do not like me

A. No B, very light C, moderate D, heavy E, severe

1. Doing things must be done slowly to ensure accuracy

A. No B, very light C, moderate D, heavy E, severe

1. My heartbeat is very strong

A. No B, very light C, moderate D, heavy E, severe

1. Nausea or discomfort in the stomach

A. No B, very light C, moderate D, heavy E, severe

1. Feeling inferior to others

A. No B, very light C, moderate D, heavy E, severe

1. Muscle soreness

A. No B, very light C, moderate D, heavy E, severe

1. I feel like someone is monitoring me and talking about me

A. No B, very light C, moderate D, heavy E, severe

1. Difficulty falling asleep

A. No B, very light C, moderate D, heavy E, severe

1. Doing things requires repeated checks

A. No B, very light C, moderate D, heavy E, severe

1. Difficult to make a decision

A. No B, very light C, moderate D, heavy E, severe

1. Afraid of taking trams, buses, subways, or trains

A. No B, very light C, moderate D, heavy E, severe

1. Difficulty breathing

A. No B, very light C, moderate D, heavy E, severe

1. Intermittent chills or fever

A. No B, very light C, moderate D, heavy E, severe

1. Avoiding certain things, occasions, or activities due to feeling scared

A. No B, very light C, moderate D, heavy E, severe

1. My mind has become empty

A. No B, very light C, moderate D, heavy E, severe

1. Numbness or stinging sensation in the body

A. No B, very light C, moderate D, heavy E, severe

1. There is a feeling of obstruction in the throat

A. No B, very light C, moderate D, heavy E, severe

1. Feeling hopeless about the future

A. No B, very light C, moderate D, heavy E, severe

1. Unable to concentrate

A. No B, very light C, moderate D, heavy E, severe

1. Feeling weak and powerless in a certain part of the body

A. No B, very light C, moderate D, heavy E, severe

1. Feeling nervous or easily nervous

A. No B, very light C, moderate D, heavy E, severe

1. Feeling heavy on hands or feet

A. No B, very light C, moderate D, heavy E, severe

1. Thinking about death

A. No B, very light C, moderate D, heavy E, severe

1. Eating too much

A. No B, very light C, moderate D, heavy E, severe

1. Feeling uncomfortable when others look at me or talk about me

A. No B, very light C, moderate D, heavy E, severe

1. There are some ideas that do not belong to you

A. No B, very light C, moderate D, heavy E, severe

1. The impulse to hit or harm others

A. No B, very light C, moderate D, heavy E, severe

1. Waking up too early

A. No B, very light C, moderate D, heavy E, severe

1. Must wash hands repeatedly, count points, or touch certain things

A. No B, very light C, moderate D, heavy E, severe

1. Not sleeping steadily or deeply

A. No B, very light C, moderate D, heavy E, severe

1. The impulse to break or destroy something

A. No B, very light C, moderate D, heavy E, severe

1. There are some ideas or thoughts that others do not have

A. No B, very light C, moderate D, heavy E, severe

1. Feeling neurotic towards others

A. No B, very light C, moderate D, heavy E, severe

1. Feeling uncomfortable in crowded places such as shops or cinemas

A. No B, very light C, moderate D, heavy E, severe

1. Feeling that everything is difficult

A. No B, very light C, moderate D, heavy E, severe

1. Waves of fear or terror

A. No B, very light C, moderate D, heavy E, severe

1. Feeling uncomfortable eating in public places

A. No B, very light C, moderate D, heavy E, severe

1. Frequently argue with others

A. No B, very light C, moderate D, heavy E, severe

1. I feel nervous when alone

A. No B, very light C, moderate D, heavy E, severe

1. Others did not make appropriate evaluations of my grades

A. No B, very light C, moderate D, heavy E, severe

1. I feel lonely even when I'm with others

A. No B, very light C, moderate D, heavy E, severe

1. Feeling restless and unsettled while sitting

A. No B, very light C, moderate D, heavy E, severe

1. Feeling worthless

A. No B, very light C, moderate D, heavy E, severe

1. Something that feels familiar becomes unfamiliar or doesn't seem real

A. No B, very light C, moderate D, heavy E, severe

1. Shouting or throwing things

A. No B, very light C, moderate D, heavy E, severe

1. Afraid of fainting in public places

A. No B, very light C, moderate D, heavy E, severe

1. I feel like others are trying to take advantage of me

A. No B, very light C, moderate D, heavy E, severe

1. I am troubled by some thoughts about 'sex'

A. No B, very light C, moderate D, heavy E, severe

1. I think one should be punished for their own mistakes

A. No B, very light C, moderate D, heavy E, severe

1. Feeling the need to finish the task quickly

A. No B, very light C, moderate D, heavy E, severe

1. I feel that my body has serious problems

A. No B, very light C, moderate D, heavy E, severe

1. I have never felt very close to anyone else

A. No B, very light C, moderate D, heavy E, severe

1. Feeling guilty

A. No B, very light C, moderate D, heavy E, severe

1. Feeling that there is something wrong with my brain

A. No B, very light C, moderate D, heavy E, severe
